# The gut-brain axis and beyond: Microbiome control of spinal cord injury pain in humans and rodents

**DOI:** 10.1016/j.ynpai.2020.100059

**Published:** 2020-12-18

**Authors:** Courtney A. Bannerman, Katya Douchant, Prameet M. Sheth, Nader Ghasemlou

**Affiliations:** a Department of Biomedical and Molecular Sciences, Queen’s University, Kingston, Ontario, Canada; b Department of Pathology and Molecular Medicine, Queen’s University, Kingston, Ontario, Canada; c Division of Microbiology, Kingston Health Sciences Centre, Kingston, Ontario, Canada; d Gastrointestinal Disease Research Unit, Kingston Health Sciences Center, Kingston, Ontario, Canada; e Department of Anesthesiology and Perioperative Medicine, Kingston Health Sciences Centre, Kingston, Ontario, Canada; f Centre for Neuroscience Studies, Queen’s University, Kingston, Ontario, Canada

**Keywords:** Chronic pain, Neuropathic pain, Neuroimmune interactions, Neuroinflammation, Neurogenic bladder, Short-chain fatty acids

## Abstract

Spinal cord injury (SCI) is a devastating injury to the central nervous system in which 60 to 80% of patients experience chronic pain. Unfortunately, this pain is notoriously difficult to treat, with few effective options currently available. Patients are also commonly faced with various compounding injuries and medical challenges, often requiring frequent hospitalization and antibiotic treatment. Change in the gut microbiome from the “normal” state to one of imbalance, referred to as gut dysbiosis, has been found in both patients and rodent models following SCI. Similarities exist in the bacterial changes observed after SCI and other diseases with chronic pain as an outcome. These changes cause a shift in the regulation of inflammation, causing immune cell activation and secretion of inflammatory mediators that likely contribute to the generation/maintenance of SCI pain. Therefore, correcting gut dysbiosis may be used as a tool towards providing patients with effective pain management and improved quality of life.

## Highlights

• Spinal cord injury often results in gut dysbiosis, in both humans and rodent models of disease.
• Shifts in gut microbiota results in a marked decrease in butyrate-producing bacteria.
• This dysbiosis, along with the “standard of care” use of antibiotics, can negatively affect recovery.
• The genito-urinary microbiome is also altered in SCI and may contribute to neurogenic bladder.
• Correcting the microbiome can result in improved recovery and neuroprotection.

## 1 Introduction

Spinal cord injury (SCI) is a devastating injury affecting approximately 2.5 million people worldwide (Kopp et al., 2013). While direct damage is restricted to the central nervous system (CNS), people often experience adverse effects in multiple organ systems that can depend on injury level and severity. For example, SCI patients have a significantly higher risk of developing cardiovascular disease and blood pressure irregularities (Nash and Gater, 2020), renal deterioration (Romo et al., 2018), and bowel and urinary tract dysfunction (Nseyo and Santiago-Lastra, 2017, Bernardi et al., 2020), all of which can result in life-threatening outcomes. These changes in the digestive and urinary systems are particularly severe and include bowel obstruction, gastrointestinal (GI) hemorrhage, oesophagitis, gastric ulcers, cholecystitis, and lithiasis (Karlsson, 2006, Pan et al., 2014, Holmes and Blanke, 2019). Approximately 11% of SCI patient rehospitalization is due to GI issues (Middleton et al., 2004, Jaglal et al., 2009).

Bacterial pneumonia and septicemia are among the leading causes of death after SCI (DeVivo et al., 1989, Soden et al., 2000) and are known to impair functional recovery (Failli et al., 2012). The majority of SCI patients receive multiple rounds of antibiotic treatment after injury due to secondary bacterial infections and urinary tract infections, leading to an increased risk of antibiotic resistance (Mylotte et al., 2000, Jahromi et al., 2014) and adversely affecting the GI microbiota (GIM). Alternatively, several host immune markers, including decreases in CD14^+^ monocytes, CD3^+^ T lymphocytes, MHC class II cells, and CD19^+^ B lymphocytes in the circulatory system (Riegger et al., 2009) have been documented, suggesting that SCI results in imbalances in both the GIM and the host immune system. This reduction in immune cells may be dependent on injury level and completeness and/or severity, with a more detrimental effect in more severe injuries and those located at higher levels of the spinal cord (Hong et al., 2019).

The gut microbiota, a term used to describe the collection of microorganisms residing in the human GI system, plays a central role in overall health, acting on cardiovascular health, digestion, nutrient absorption, training/maintenance of the mucosal immune system (Shi et al., 2017, Gorkiewicz and Moschen, 2018). Dysbiosis, the development of imbalance in the GIM, correlates with pronounced systemic and localized effects on the host, including increased inflammation, decreased functional recovery, and increased pain or hypersensitivity. The interplay between the GIM and the host immune system contributes to immune cell maturation and activity (Rizzetto et al., 2018). Dysbiosis of the GIM can result in changes in the expression of immune cell-derived mediators, which in turn can alter the immune response (Rosser and Mauri, 2016). The GIM plays an essential role in the regulation of both the innate and adaptive immune systems (Belkaid and Harrison, 2017) by not only providing feedback, but also in the development of the immune system in neonates and the functional tuning throughout life. The production of various compounds by the GIM, such as short-chain fatty acids (SCFAs) highlighted in this review, also have the ability to influence the immune system within the GI tract (Chang et al., 2014) and also in peripheral tissues where they promote the release of immune cells into the bloodstream (Clarke et al., 2010, Belkaid and Harrison, 2017).

**Fig. 1:**
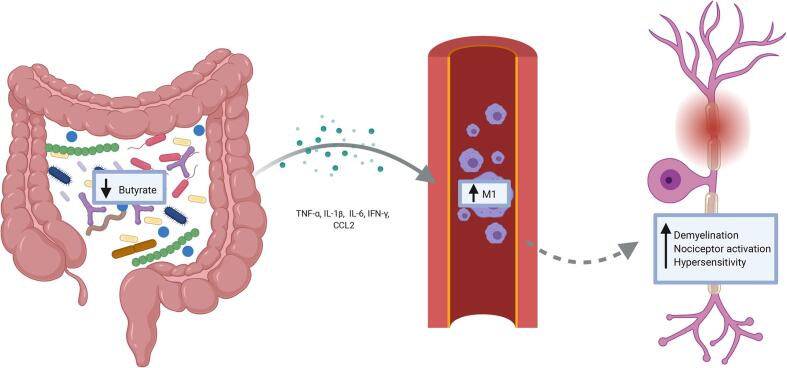
A decrease in butyrate-producing organisms in the gastrointestinal tract is observed after a spinal cord injury. There is also an increase in TNF-α, IL-β, IL-6, IFN-γ, and CCL2 from activated immune cells and colonic tissue. This increase in inflammatory cytokines pushes macrophages to an M1 phenotype, which has been shown to result in increased demyelination, nociceptor activation, and hypersensitivity.

The potential interplay between altered immune cell activation and gut dysbiosis have been suggested to contribute to the development of chronic pain in various diseases, including cancer pain (Shen et al., 2017), multiple sclerosis (Mirza and Mao-Draayer, 2017), fibromyalgia (Clos-Garcia et al., 2019), and irritable bowel syndrome (IBS) (Jeffery et al., 2012, Hugerth et al., 2020). Many of these diseases include damage to the CNS and a strong pain phenotype. This chronic pain is often coupled with depression and a decreased quality of life (Kennedy and Hasson, 2017, Muller et al., 2017). Here, we explore how changes in the GIM may play a role in changes to the neuroimmune response both peripherally and centrally, and, consequently, the development of chronic pain after SCI (see Fig. 1).

## 2 Spinal cord injury pain in humans

Pain is categorized as having either a neuropathic (pain developing from damage to the somatosensory nervous system that does not diminish as the body heals), inflammatory (pain caused directly by tissue damage and inflammation, which is most often acute but can become chronic), or nociceptive (pain that occurs in response to immediate tissue damage and which serves as an early-warning protective response) origin (Woolf, 2010, Pinho-Ribeiro et al., 2017). Nociceptive pain is common after SCI and is primarily due to the overuse of the upper body, muscle weakness, poor posture, and spasticity (Bryce et al., 2012). Approximately 60–80% of SCI patients will experience chronic pain after their injury (van Gorp et al., 2015). While conventional pharmaceutical means such as opioids (e.g., tramadol, morphine, oxycodone), nonsteroidal anti-inflammatories (e.g., aspirin, ibuprofen), and anticonvulsants (e.g., gabapentin, pregabalin) are frequently used to aid those suffering from SCI pain, this pain is often refractory to treatment (Mehta et al., 2016). More than half of SCI patients will develop neuropathic pain (Burke et al., 2017), which is particularly challenging to treat, given that the mechanisms behind its development are poorly understood. Neuropathic pain is felt either at-level (meaning, felt at the spinal injury level and up to 3 segments rostral) and below-level (below the level of injury) (Bryce et al., 2012). Typically, at-level pain occurs early after injury, while below-level pain develops in the more chronic stages – months or years post-injury (Siddall et al., 1997, Siddall et al., 2003). Below-level pain after partial interruption of axonal tracts has been attributed to a loss of descending inhibition on below-level nociceptive pathways, such that activity in surviving ascending pain pathways is increased (Bruce et al., 2002, Hains et al., 2002, You et al., 2008). At-level pain is typically due to damage to the spinal roots as well as to the cord itself, whereas below-level pain is due only to spinal cord damage (Siddall et al., 2003). The majority of patients with at- and below-level pain experience spontaneous neuropathic pain (Finnerup et al., 2014).

Most spinal cord injuries occur in the cervical region (C1 to C7-T1) of the cord (Sekhon and Fehlings, 2001). Siddall and colleagues found that a higher proportion of patients with injuries caudal to or at the cervical region experienced below-level neuropathic pain than those with SCI rostral to the cervical region of the cord (Siddall et al., 2003). In addition to location, a positive correlation is observed between the prevalence of below-level pain and the severity of injury (Kolstadbraaten et al., 2019). It is also important to note that SCI rarely happens in the absence of other injuries. It is often accompanied by a traumatic brain or peripheral nerve injury, or in the case of motor vehicle accidents with severe trauma to critical organs, which can complicate the presentation of pain phenotypes.

After the spinal cord is injured, it can take patients several years to recover, if at all, and the degree of recovery can be minimal in some cases (Fakhoury, 2015). In a study of 265 SCI patients, it was observed that over 80% of patients remained within two sensory levels – defined as the lowest spinal cord level that still has normal touch sensation – one year after their injury (Lee et al., 2016). Spontaneous recovery in humans often does not reach a plateau until approximately 6–12 months after injury (Kjell and Olson, 2016). Several surveys have found that people with SCI often place the return of sexual, bladder, and bowel function and the reduction of chronic pain as key quality-of-life concerns (Ku, 2006, Sezer et al., 2015, Burke et al., 2018). A challenge that comes along with SCI research and assessing functional sensory recovery is that SCI is incredibly heterogeneous. Thus, a concern among the research community is that limited functional recoveries might be challenging to detect. Although individual patient recovery scores may only improve to a small degree, any change is likely to be meaningful to patients and may represent a higher degree of independence (Wu et al., 2015).

## 3 Rodent models of spinal cord injury pain

SCI is usually modelled in the laboratory using rodents (rats and mice) due to cost, ease of care, and somewhat similar pathologies to the human response to injury. While rodent models of SCI have some notable differences to humans, such as the proliferation of cells at the site of injury (Ma et al., 2001, Goritz et al., 2011) and the lack of the development of fluid-filled cysts in the mouse (Fleming et al., 2006), they provide a consistent and cost-effective strategy to study SCI with the goal of translating findings to the clinic. Most SCI studies are performed in female rats or mice due to ease of bladder expression after injury. While male and female rats exhibit similar pain profiles, locomotor recovery, and microglial/astrocytic reactivity after SCI (Gaudet et al., 2017, Gaudet et al., 2018, Walker et al., 2019), myelin sparing is slightly greater in female rats than in males (Walker et al., 2019). In mice, males recovered more frequent hindlimb stepping than female mice, with slightly increased lesion length (McFarlane et al., 2020). Female mice, on the other hand, exhibit increased anxiety scores using the light–dark box test and the elevated plus-maze (Fukutoku et al., 2020).

SCI is modelled using various methods, including hemisection, complete transection, clip-compression, or contusion/compression of the spinal cord, with each suited to address specific biological questions. In humans, the majority of SCI cases are due to motor vehicle accidents, falls, and violence (DeVivo and Chen, 2011); these types of injuries most closely resemble those produced using the contusive model. Several tools exist to carry out contusion injury in rodents, including the Ohio State University electromagnetic SCI device (Behrmann et al., 1992, Jakeman et al., 2000), the Infinite Horizon Impactor (Scheff et al., 2003, Ghasemlou et al., 2005), and the New York University Impactor (Gruner, 1992, Kuhn and Wrathall, 1998). There are several recent reviews on rodent models of SCI (Cheriyan et al., 2014, Steward and Willenberg, 2017, Ahmed et al., 2019, Alizadeh et al., 2019). Most injuries performed in rodents are contusion injuries, and most often in the thoracic region of the spinal cord (Kramer et al., 2017). Many laboratories are also now using cervical injury, where most human injuries occur (Sekhon and Fehlings, 2001).

Pain has been defined by the International Association for the Study of Pain (IASP) as *“an unpleasant sensory and emotional experience associated with actual or potential tissue damage, or described in terms of such damage,”* while the newly proposed definition would be “*an aversive sensory and emotional experience typically caused by, or resembling that caused by, actual or potential tissue injury”* (Raja et al., 2020). Given the difficulty in measuring emotional experience in the rodent, the terms allodynia (pain from a normally non-noxious stimulus), hyperalgesia (increased pain from a normally noxious stimulus), and hypersensitivity (increased response to a stimulus) are often used instead. Changes in responses to mechanical, cold, and heat stimulus are measured in the laboratory using various tools, including von Frey monofilaments (mechanical), the acetone test (cold), and the hot plate or Hargreaves radiant heat tests (thermal heat). Several groups have found injury severity as a critical modulator of the mechanical and thermal behavioural response to SCI in rodents (Lindsey et al., 2000, Boadas-Vaello et al., 2018), as well as in a single study in patients (Marcondes et al., 2016).

Several groups have sought to provide a detailed characterization of the development and maintenance of the hypersensitivity response after SCI, while others have provided insight into changes at specific timepoints (Bruce et al., 2002). In these studies, mechanical hypersensitivity is most often observed at 14 days post-injury (dpi) but can develop as early as 7 dpi or late as 28 dpi depending on injury severity, strain, and model used (Kerr and David, 2007, Watanabe et al., 2015, Fandel et al., 2016, Martini et al., 2016, Qian et al., 2017, Tateda et al., 2017, Shin et al., 2018, Li et al., 2019, Wang et al., 2019). The emergence of thermal heat hypersensitivity shows more variability but is found to start between 7 and 21 dpi (Meisner et al., 2010, Chen et al., 2012, Falnikar et al., 2016, Nees et al., 2016, Castany et al., 2018, Liu et al., 2019). Recent work from our laboratory has found that moderate (50kdyn) contusion injury with 60 s of sustained spinal cord compression results in an earlier onset of thermal heat and mechanical hypersensitivity when compared to a moderate contusion injury without compression of the spinal cord. Sustained compression of the spinal cord results in increased neuroinflammation and reduced white matter sparing, which have been correlated with the development of hypersensitivity in mouse models of SCI (Lindsey et al., 2000, Hoschouer et al., 2010, Boadas-Vaello et al., 2018, Park et al., 2018). Connections between pain and the immune response have been recently identified and have been reviewed by others (Scholz and Woolf, 2007, Ren and Dubner, 2010, Verma et al., 2015, Raoof et al., 2018); these are discussed only briefly here. Work from several groups is starting to unravel the mechanisms underlying the interaction between the nervous and immune systems in SCI pain (Bastien and Lacroix, 2014, Lerch et al., 2014, Matyas et al., 2017, Aceves et al., 2019, Chhaya et al., 2019). Immune cells that can contribute to the pain response include circulatory myeloid and lymphoid cells and tissue‐resident cells.

Microglia proliferate extensively during the first week after injury, and form a scar in the weeks following injury with astrocytes and infiltrating macrophages (Bellver-Landete et al., 2019). This microglial response leads to the recruitment of circulatory immune cells to the site of injury through the release of various cytokines, such as IL-1β and TNF-α; the mRNA of these cytokines can be detected within 1–12 h after injury in mice (Pineau and Lacroix, 2007), while secretion of IL-1β, IL-6, and TNF-α has been detected from microglia as early as 5 h after injury in patients (Yang et al., 2004). Neutrophils are the first circulatory cells to infiltrate the spinal cord and can remain in the cord for at least 72 h post-injury (Beck et al., 2010). Dampened neutrophil trafficking to the spinal cord results in a reduction of IL-6, TNF-α, and IL-1β at 12 h post-injury, and leads to improved locomotor recovery (Saiwai et al., 2010). These same cytokines have all been implicated in pain development (Leung and Cahill, 2010, Guptarak et al., 2013, Xiang et al., 2019).

Following the infiltration of neutrophils, circulating monocytes are recruited to the spinal cord and are likely persist indefinitely in the cord as macrophages (Donnelly and Popovich, 2008). These macrophages, which can consist of both microglia and monocytes, exist in a spectrum of pro- and anti-inflammatory states (previously referred to as M1 and M2). Flow cytometric analysis suggests that once macrophages phagocytose myelin, they shift to an anti-inflammatory phenotype (Boven et al., 2006). In contrast, the phagocytosis of iron, through the phagocytosis of red blood cells, shifts them towards a more pro-inflammatory state (Kroner et al., 2014). Mice with reduced numbers of proinflammatory macrophages have significantly less thermal heat and mechanical hypersensitivity 2 weeks after SCI (Honjoh et al., 2019). After injury, the role of macrophages is not purely detrimental. Recent work has demonstrated that peripheral macrophages help control microglia activation, preventing acute and chronic inflammation in the injured spinal cord (Greenhalgh et al., 2018), and the contribution of these cells to repair and revascularization in SCI has also been well-characterized (David et al., 2015, Gensel and Zhang, 2015, Wynn and Vannella, 2016, Orr and Gensel, 2018).

Finally, T and B lymphocytes infiltrate the spinal cord 2–3 weeks after injury, depending on the species or rodent strains used (Sroga et al., 2003). The role of T cells in SCI is less clear than that of myeloid cells, though there is evidence that they can modulate macrophage function, microvascular endothelial integrity, and axonal conduction (Yarom et al., 1983, Naparstek et al., 1984, Janeway et al., 1988). For example, CD4 + T cells secrete immune mediators or express factors implicated in immune cell recruitment including IL23, IL23R, IFN-γ, CXCL9, CXCL11, and iNOS after SCI (Li et al., 2018). A reduction of CD4 + T cells in the spinal cord of mice following injury has also been associated with a reduced risk of developing thermal hypersensitivity (Li et al., 2018). While only CD4^+^ T cells have been shown to have a role in SCI pain at this time, both CD8^+^ and γδ T cells have the potential also to be involved in the response to injury (Sun et al., 2018, Liu et al., 2019).

## 4 The microbiome in health and disease

The bacterial population inhabiting the GI tract, called the GIM, is made up of trillions of organisms and are emerging as critical players in both local and systemic host processes (Gill et al., 2006). The microbiota composition varies according to the region of the GI tract, with gram-positive facultative anaerobes predominating in the proximal small intestine, gram-negative anaerobes in the distal small intestine, and obligate anaerobes in the colon (Chichlowski and Rudolph, 2015). Bacteria within the GIM contain a repertoire of enzymes that vastly outnumber those in their host. Therefore, a significant contribution to host health is the ability to metabolize nutrients that the host cannot (Oliphant and Allen-Vercoe, 2019). Once nutrient substrates reach the GIM, they are utilized by bacteria as carbon sources resulting in the production of bacterial metabolites, such as SCFAs, which are the most abundant microbial end-product, contributing to approximately 10% of the caloric content required by the host (den Besten et al., 2013). The SCFAs acetate, propionate, and butyrate have well-studied and vital physiological functions in the host, such as providing energy to intestinal epithelial cells and regulating inflammatory responses (Morrison and Preston, 2016).

The degradation of specific molecules produces other bacterial metabolites; for example, aromatic amino acid degradation can result in compounds that act as toxins or neurotransmitters (Oliphant and Allen-Vercoe, 2019). The catabolism of tryptophan yields tryptamine and indoles, where tryptamine not only plays a role in regulating intestinal motility and immune function but can also potentiate the inhibitory response of cells to serotonin (Takaki et al., 1985, Gao et al., 2018). Furthermore, basic amino acid fermentation can yield biogenic amines which act as neurotransmitters (Carabotti et al., 2015). Therefore, the composition of the microbiota, as well as their microbial end-products, are critical for mediating several processes within the host.

The GIM forms a symbiotic relationship with the host, and dysbiosis leads to changes in the microbial community structure that have been associated with several host pathologies (Guarner and Malagelada, 2003). One main sign of dysbiosis is a loss in overall bacterial diversity. As such, diversity measures are frequently used in microbiome studies to analyze community membership. A common approach to assessing community diversity is by measuring changes in alpha and beta diversity within and between samples (Wagner et al., 2018). Alpha diversity measures intrasample richness, which is how many different species are detected in a microbial community, and evenness refers to how similar taxa abundance levels are in a community (Wagner et al., 2018). Beta diversity represents the similarity or distance (dissimilarity) between each pair of samples by comparing microbial communities based on their composition (Wagner et al., 2018). Beta diversity allows researchers to explore the rate of change in species composition or bacterial turnover from one community to another (Goodrich et al., 2014). The concept of community diversity is vital for human health since several pathologies correlate with decreased microbiome diversity, as certain species overgrow or are depleted during dysbiosis.

## 5 Bacterial changes in the gut microbiome of patients after a spinal cord injury

Bacteria can be classified and subdivided into ever more specific categories using taxonomic ranks. Bacteria are classified as a kingdom; within this kingdom exists many phyla. Two phyla that are a focus of this review are Bacteroidetes and Firmicutes. Classification of bacteria populations has become increasingly more specific with ever greater members of each phyla discovered, providing richer details in class, order, family, genus, and species of bacteria. Most microbiome studies cited in this review evaluate changes at the phylum, class and order level.

Microbiota changes after an injury have been documented in both mice and humans (Kigerl et al., 2018, Kigerl et al., 2020, Wallace et al., 2019); see Supplemental Table 1 for a list of all SCI microbiome studies (as of August 2020) and their major findings. Studies show that the diversity of the gut microbiome after injury was drastically reduced, along with a change in the gut structural composition (Zhang et al., 2018, Zhang et al., 2019). One study found an increase in the abundance of bacteria within the families *Veillonellaceae* and *Prevotellaceae* and a decrease of *Bacteroidaceae* and *Bacteroides* after SCI. The amount of *Bacteroidaceae* and *Bacteroides* in the quadriplegia group and *Acidaminococcaceae*, *Blautia*, *Porphyromonadaceae*, and *Lachnoclostridium* in the paraplegia group were significantly increased after injury (Zhang et al., 2018); while bowel dysfunction is not related to the neurological level of SCI or injury severity (Han et al., 1998), changes in GIM may be affected. At the phylum level, there was a significant decrease in Firmicutes in SCI patients (Zhang et al., 2019). In another similar study looking at only quadriplegic patients, *Bacteroides* and *Blautia* were significantly more abundant in patients than in healthy subjects, along with a decrease in *Faecalibacterium* and *Megamonas.* Although not all of these bacteria have been investigated for their role in pain, a decrease in *Faecalibacterium* numbers has been associated with worse pain scores in myalgic encephalomyelitis/chronic fatigue syndrome patients (Nagy-Szakal et al., 2017).

**Table 1:** summary of all SCI microbiome studies.

Species	Microbiome location	Injury type	# of patients	Increased bacteria populations	Decreased bacteria populations	Conclusions	Reference
Mouse	GI	Contusion at T9	6–10 (Female C57BL/6)	Clostridiales	Bacteriodales	GD impairs functional recovery, correcting the microbiome results in improved locomotion	(Kigerl et al., 2016)
Mouse	GI	Contusion at T9	6 (Female C57BL/6)	Bacteroidetes, Proteobacteria	Firmicutes	Activated astrocytes, and CD11b + cells in the SC correlated with increased GD	(Myers et al., 2019)
Mouse	GI	Contusion at T10	4 (Female C57BL/6)	Bacteroidetes, Clostridiales	Firmicutes, Lactobacillales, Bifidobacteriales	Melatonin helps correct GD, improving locomotion and reducing CCL2, IL-17, INF-γ	(Jing et al., 2019)
Rat	GI	Contusion at T10	8 (Female)	*Bifidobacteriaceae, Clostridiaceae*	*Clostridium saccharogumia*	Levels of IL-1β, IL-12, and MIP-2 correlate with GD	(O'Connor et al., 2018)
Rat	GI	Contusion at C5	Female Lewis	Dysbiosis was observed but exact changing populations not reported		Fecal transplants improve anxiety	(Schmidt et al., 2020)
Human	GI	Complete T6 or above, or Traumatic cauda equina syndrome	30 (Male and female)	None reported	*Pseudobutyrivibrio*, *Dialister*, *Megamonas*	Butyrate-producing bacteria are significantly reduced	(Gungor et al., 2016)
Human	GI	Chronic traumatic complete SCI	43 (Male)	*Bacteroides*, *Blautia, Lachnoclostridium, Escherichia shigella*	*Megamonas, Dialister, Subdoligranulum*	GD correlates with neurogenic bowel dysfunction	(Zhang et al., 2018)
Human	GI	Complete C3 to C8	20 (Male)	Proteobacteria, Verrucomicrobia, *Bacteroides*, *Blautia*, *Escherichia Shigella*, *Lactobacillus*, *Akkermansia*	Firmicutes, *Faecalibacterium, Megamonas, Dialister, subdoligranulum*	GD correlates with changes in lipid metabolism	(Zhang et al., 2019)
Human	GI	Cervical to lumbar	32 (Male and female)	*Erysipelotrichaceae, Acidaminococcaceae, Rikenellaceae, Lachnoclostridium, Eisenbergiella, Alistipes, Oscillibacter, Anaerotruncus*	*Campylobacter*, *Burkholderiaceae*	Microbiome can differ depending time after SCI	(Li, 2020)
Human	GI	Cervical to lumbar, both complete and incomplete	23 (Male and female)	*Parabacteroides, Alistipes, Phascolarctobacterium, Christensenella, Barnesiella, Holdemania, Eggerthella, Intestinimonas, Gordonibacter, Bilophila, Flavonifractor,* and *Coprobacillus*	*Haemophilus, Clostridium sensu stricto 1, Veillonella, Dialister, Roseburia, Megamonus, Leuconostoc, Lachnospira, Megasphaera, Rhodococcus, Ruminococcus, Subdoligranulum, Pesudobeautyrivibrio*, and *Faecalibacterium*	GD occurs after SCI	(Lin et al., 2020)
Human	Urinary	Complete T4	1 (Male)	Baseline or control measurements not taken		Bacteria may be found in urine	(Nally, 2018)
Human	Urinary	C3-C6, ASIA A/B	3 (Male)	Proteobacteria and Firmicutes were the dominate bacteria type		Probiotic treatment effects are transient	(Bossa et al., 2017)
Human	Urinary	Not reported	27 (Male and female)	*Klebsiella* (male), *Escherichia* (female), *Enterococcus* (female)	*Lactobacillus*, *Corynebacterium*, *Staphylococcus* (male), *Streptococcus* (male), *Prevotella* (female)	Different catheterization methods can alter microbiome	(Fouts et al., 2012)
Human	Urinary	Not reported	45 (Male and female)	*E. coli* and *Klebsiella* seen at study start		Weekly oral cyclic antibiotics are effective at reducing UTIs	(Dinh et al., 2019)
Human	Urinary	Cervical to lumbar	5 (Male)	*Pseudomonas* and *E. coli* are dominant bacteria types		Probiotic instillation is well-tolerated	(Forster et al., 2019)
Human	Vaginal	Tetraplegic or paraplegic	52 (Female)	*E. coli, Corynebacterium*	*Lactobacillus*	SCI alters vaginal bacteria populations	(Pires, 2016)

GI = Gastrointestinal, GD = Gut dysbiosis, SC = Spinal cord.

A study subdividing patients into upper motor neuron (complete injuries C4-T5) and lower motor neuron (complete T1-L2) injuries found that *Pseudobutyrivibrio*, *Dialister,* and *Megamonas* genera were significantly lower in the upper motor neuron group than in healthy controls (Gungor et al., 2016). Meanwhile, *Roseburia*, *Pseudobutyrivibrio,* and *Megamonas* genera were significantly reduced in the lower motor neuron group. Between the two groups, there was significantly less *Marvinbryantia* genus in the upper motor neuron group than in the lower motor neuron group; similar trends have been observed in a recent study of SCI patients (Lin et al., 2020). Overall, these findings suggest a marked difference between studies investigating changes in the microbiome after injury, which is likely due to the high variability that exists between people. It is also important to note that post-injury SCI patients have a high prevalence of multi-drug resistant organisms colonization or infection due to extended hospital stays and increased exposure to antibiotics. A retrospective analysis found that 43% of acute SCI patients were either colonized or infected with multi-drug resistant organisms, with methicillin-resistant *Staphylococcus aureus* being the most common in 34% of patients (Toh et al., 2020).

An essential role of a healthy microbiome is preventing the colonization of the gut by pathogenic bacteria. With reduced microbiome diversity, these patients are at a greater risk for GI tract diseases, including *Clostridium difficile* (Dumford et al., 2011). SCI can directly impact the GI tract, with as many as 1 in 3 patients complaining of chronic GI problems that include localized abdominal pain, difficulties with bowel evacuation, abdominal distention, and GI hyperreflexia (Stone et al., 1990, Nielsen et al., 2017). Furthermore, almost 1 in 4 require at least one admission to the hospital for a GI complaint following their injury (Stone et al., 1990). The pathophysiology of GI pain in SCI patients is still not completely understood; however, emerging evidence suggests GI resident bacteria may play a role in modulating pain (Moloney et al., 2016, Arora et al., 2017, Russo et al., 2018, Guo et al., 2019).

## 6 Bacterial changes in the gut microbiome of laboratory rodents after a spinal cord injury

**Fig. 2:**
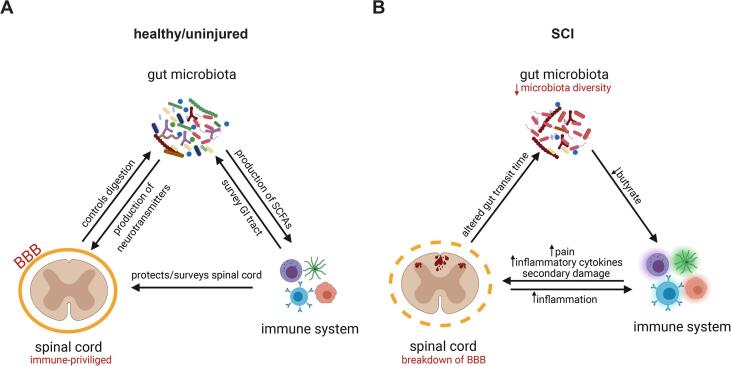
The microbiome, immune system, and nervous system intensively interact with each other in both conditions of health and disease. The naïve mouse the spinal cord (A) is considered to be an “immune privileged” site, protected by the blood brain barrier (BBB). The central nervous system helps control digestion and the gut microbiota plays an important role in the production of neurotransmitters and short-chain fatty acids (SCFAs). The immune system surveys and protects both the spinal cord and gastrointestinal tract. There is a breakdown of the BBB after spinal cord injury (B), allowing for the influx of neutrophils, lymphocytes, and macrophages, as well as the activation of microglia. These cells will produce various inflammatory mediators that will not only act upon the other immune cells but also the central nervous system, causing increased inflammation and pain. The altered expression of SCFAs, vitamins, and neurotransmitters in the gut can in turn influence the immune and nervous systems.

The Popovich group from Ohio State University was the first to study the contribution of the microbiome in SCI, showing that the microbiome plays an important role in functional recovery after injury (Kigerl et al., 2016, Schmidt et al., 2020). There is, therefore, a potential link between the GIM, the gut, and the brain, modulating visceral pain within the GI mucosa (see Fig. 2). This complex interaction is mediated locally through endocrine or immune activity, and systemically through the production of bacterial metabolites, including SCFAs vitamins such as folate and biotin. It is also mediated by neuroactive metabolites like serotonin and gamma-butyric acid (Sharon, 2014; Chichlowski and Rudolph, 2015). Various environmental stressors, such as the use of antibiotics, advancing age, or trauma to the GI tract, can disrupt the relationship between the GIM, the gut, and the brain and alter the composition of the microbiota as well as visceral pain. The use of rodents allows researchers to study the effects of altering the microbiome on cellular and molecular outcomes, such as immune cell activation or demyelination in the spinal cord. Mice show an increase in the abundance of Bacteroidetes and a decrease in Firmicutes after injury. Rodents who received a severe contusion injury showed a significant decrease in the relative abundance of Lactobacillales and Bifidobacteriales and an increase of Clostridiales (Kigerl et al., 2016, O'Connor et al., 2018, Jing et al., 2019). Myers et al. also saw a decrease in Firmicutes with an increase in Bacteroidetes and Proteobacteria at 42 days post-SCI (Myers et al., 2019). These microbiome changes can be seen very early after injury, often as early as 3–7 dpi (Myers et al., 2019, Schmidt et al., 2020).

Gut vascular permeability, the ability for substances or cells to move in and out of the GI tract, is an essential aspect of gut health and directly impacts the GIM composition. GI vascular permeability in adult male C57BL6 mice who received a moderate contusion at T9 is increased in the acute phase (48 hr) after injury and maintained into the chronic phase (4 weeks) (Herrera et al., 2020). It has been observed that the GI mucosal tissue was compromised as early as 3dpi though the resulting damage was repaired by 3 weeks after a 300-kdyn impact (15 s dwell) at T3 in male Wistar rats. There is also evidence of reduced blood flow to the GI tract early after SCI in rats fed a liquid nutrient meal (Besecker et al., 2017). Interestingly, male Wistar rats with a contusive 300-kdyn SCI at T3 showed a reduction of gut emptying starting at 3dpi that diminished until at least 3 weeks post-injury (Qualls-Creekmore et al., 2010). Acutely after injury, there is an increase in the gastric expression of the inflammatory cytokine transcripts ICAM1 and CCL3 (Besecker et al., 2017), with an increase in IL-17, IFN-γ, CCL2, and IL-1β measured from colonic tissues at more chronic stages of injury (Jing et al., 2019). The production of these inflammatory cytokines could potentially play a role in the chronic activation of the immune system, increased secondary damage, and negatively impact functional outcomes (see Fig. 1).

Due to the comorbid injuries also seen with SCI, such as frequent UTIs, the inability to completely void their bladder, and a reduced ability to fight off infections, many patients are often overprescribed antibiotics (Brommer et al., 2016, Clarke et al., 2020, Guilcher et al., 2020). The effects of antibiotics on the diversity of the GIM have been well documented and reviewed (Guarner and Malagelada, 2003, Ferrer et al., 2017, Guzman-Rodriguez et al., 2018). Interestingly, SCI mice who had their GIM disrupted with antibiotics before injury show reduced locomotor recovery (Kigerl et al., 2016, Jing et al., 2019). There is, however, emerging literature pointing to the potential benefits of antibiotics, particularly minocycline, in reducing chronic pain after SCI in rats and its ability to be neuroprotective (Marchand et al., 2009, Cho et al., 2011, Aceves et al., 2019). This is due to the ability of minocycline to decrease microglial activation, TNF-α, and IL-6 expression in the spinal cord after injury (Sencar et al., 2020). Azithromycin, another antibiotic with neuroprotective and analgesic properties after SCI, has also been associated with a reduction in the proportion of proinflammatory macrophages (Gensel et al., 2019, Kopper et al., 2019). There can be a great deal of variation with the gut motility and integrity between various rodent studies; this could be due to several factors. It is not uncommon for SCI researchers to treat rodents with antibiotics after surgery, which may affect the GIM and functional outcomes. The location and severity of the injury will also significantly affect functional outcomes as well, as cervical SCI may have a more profound effect on recovery than those occurring caudally.

## 7 Contribution of the microbiome to the development of pain

Recent work from across different injury and disease models have now shown that bacteria contribute to the development and maintenance of pain. However, the microbiome itself only becomes harmful when there is a dramatic change in the proportions of bacteria. Patients with IBS commonly describe pain in their abdominal region. Although there is no clear microbiome profile that denotes an IBS patient, there is an increase in Firmicutes-associated taxa and decreased *Bacteroides*-associated taxa (Jeffery et al., 2012, Zhang et al., 2018, Hugerth et al., 2020). Microbiome analysis was also done in fibromyalgia patients living with severe chronic pain, demonstrated a similar decrease in overall microbiome diversity, specifically of *Lachnospiraceae* and Firmicutes (Clos-Garcia et al., 2019). A decrease in butyrate-producing *Bacteroides*, a subset of bacteria in the phylum Firmicutes (Fu et al., 2019), has also been observed in multiple sclerosis, which shares many similarities with SCI, including neuroinflammation, demyelination, and a majority of patients with chronic pain (Miyake et al., 2015, Chen et al., 2016a, Mirza and Mao-Draayer, 2017). Finally, the fecal matter of complex regional pain syndrome patients showed decreased microbiome diversity and Firmicutes in particular, when compared to healthy patients (Reichenberger et al., 2013). Although these diseases all have different mechanisms of action, they share reductions in *Bacteroides* and Firmicutes.

*Bacteroides* plays a generally beneficial role in GI health, fermenting indigestible polysaccharides into SCFAs, particularly acetate and propionate, which are a vital source of energy (Cummings, 1981). Like *Bacteroides*, Firmicutes are also involved in the fermentation of dietary products and the production of SCFAs SC (Duncan et al., 2007) with butyrate being the primary product (Macfarlane and Macfarlane, 2003). Associations are observed between the amount of Firmicutes and depression and anxiety states (Bangsgaard Bendtsen, 2012; Yu, 2017), which are common among those living with SCI (Craig et al., 2009). Decreased levels of Firmicutes in inflammatory bowel disease has also been associated with reduced intestinal mucosal protection function (Sokol et al., 2009), which also occurs in SCI. These SCFAs could contribute to pain by modulating the inflammatory response. Recent work investigating IBS has shown SCFAs, specifically butyrate, to be effective in reducing pain, controlling inflammation, and peripheral nerve sensitization (Kannampalli et al., 2011). Both *Bacteroides* and Firmicutes are essential in maintaining a healthy GIM and dramatic changes in either population or the substances they produce can adversely impact overall wellbeing and pain outcomes.

## 8 Neuroinflammation and the microbiome

Among bacterial products, butyrate and acetate SCFA can exert anti-inflammatory effects on macrophages (Chen et al., 2007). There appeared to be a significant decrease in the number of butyrate-producing members after SCI, potentially resulting in an unchecked pro-inflammatory population of macrophages (Ji et al., 2016). Furthermore, the central role of both butyrate and butyric acid in maintaining the patency of the GI barrier (Rios-Covian et al., 2016) suggests that a reduction in butyrate-producing bacteria may lead to reduced integrity of the intestinal barrier leading to increased microbial translocation of bacteria across the GI barrier. Translocation of bacteria and bacterial constituents result in the activation of the peripheral immune system (Brenchley et al., 2006) and with-it increased hypersensitivity (Canani et al., 2011). The *Lachnospiraceae* family, which includes *Pseudobutyrivibrio* and *Roseburia,* is decreased after injury and responsible for producing butyric acid (Meehan and Beiko, 2014). It has been found that butyrate administration decreased inflammation triggered by high-fat diets, with a notable decrease in TNF-α (Mattace Raso et al., 2013). The family of *Lactobacillales* is responsible for fermenting carbohydrates into lactic acid in the GI tract. Improved locomotor recovery, increased white matter sparing, and protective immune response in the gut-associated lymphoid tissues was seen when SCI mice were enriched with lactic acid-producing bacteria (Kigerl et al., 2016). Increased white matter sparing, among other mechanisms, has been associated with reduced hypersensitivity (Vierck, 2019).

In contrast to the protective effects offered by butyrate and butyrate-producing bacteria, an increase in gram-negative bacteria, such as Bacteroidetes, can lead to increased plasma levels of Lipopolysaccharides (LPS) and cause increased inflammation, as shown by an increase in the marker CD14 and IL-6 (Laugerette et al., 2011, Graham et al., 2015). Four inflammatory cytokines have been identified to be released from colonic tissue after an injury, IL-17, IL-1β, IFN-γ, and CCL2, some of which could be responsible for driving the immune system to a more inflammatory state. IL-17 has been shown to activate astrocytes in the spinal cord (You et al., 2017) as well as be involved with synergistic inflammatory signalling with other cytokines, including TNFα, IL-1β, IFN-γ, and CCL2 (Chabaud et al., 1998, Bastien and Lacroix, 2014, Bastien et al., 2015, Bellver-Landete et al., 2019). It may play an essential role in promoting neuroinflammation by activation of STAT3 (Zong et al., 2014). IL-1β plays a vital role in immune cell recruitment (Pineau and Lacroix, 2007). It also seems to play a role in pain as mice with impaired IL-1β signalling do not develop mechanical hypersensitivity after a spinal nerve ligation (Kleibeuker et al., 2008). IFN-γ has also been shown to aggravate spinal cord lesions and reduce locomotor recovery after injury (Sun et al., 2018) as well as polarize macrophages to a pro-inflammatory phenotype (Shapouri-Moghaddam et al., 2018). Finally, CCL2 contributes to the recruitment and activation of macrophages, T cells, and B cells. In human SCI, it has been found to increase in the cerebrospinal fluid in a severity dependent manner (Liu et al., 2005, Kwon et al., 2010).

The gut microbiome not only interacts with the immune system but also with the nervous system. Recent work has demonstrated that bacteria can, directly and indirectly, sensitize nociceptors (Chiu et al., 2013, Shen et al., 2017, Boer et al., 2019). The activation of nociceptors, either by the bacteria itself or through the products they secrete, can result in the release of neuropeptides from the peripheral terminals and can induce neurogenic inflammation (Saria, 1984, Sauer et al., 2001, Meseguer et al., 2014, Lai et al., 2020). For example, LPS is a component of the cell wall of gram-negative bacteria, including Bacteroidetes*.* An increase in LPS levels has been observed after SCI in blood plasma, most likely due to bacterial translocation from the gut (Liu et al., 2004, Zhang et al., 2019)*.* Its receptor, TLR4, is expressed by TRPV1-positive nociceptor neurons resulting in their sensitization (Wadachi and Hargreaves, 2006, Diogenes et al., 2011). TRPV1 expression has been shown to increase after SCI (Zeng et al., 2018). The sensitization of TRPV1 positive neurons by increased LPS could potentially contribute to increased hypersensitivity, with a similar mechanism being found in the onset of cancer pain (Li et al., 2015).

Bacteria can also interact directly with nociceptors to sensitize them. Work by Chiu and colleagues was among the first to show that bacteria can cause calcium flux in nociceptors and that bacterial load correlates with the onset of mechanical and thermal hypersensitivity (Chiu et al., 2013). However, nociceptor sensory neurons, specifically TRPV1^+^ neurons, can also have an immunosuppressive effect on peripheral immune cells (Baral et al., 2018). The gut contains not only a large number of peripheral immune cells but also is highly richly innervated by sensory neurons. Although inflammation after SCI is commonly thought to be detrimental, it is also necessary to help clear tissue debris in an effort to allow for regeneration to occur. SCI can result in “leaky gut”, where bacteria are more likely to translocate from the GI lumen into extra-intestinal sites, including organ spaces, or into the bloodstream (Liu et al., 2004, Kigerl et al., 2016). This increase in bacterial translocation could increase the chance of bacteria interacting with nociceptors resulting in increased sensitization.

Modulating the microbiome may provide new avenues towards the treatment of SCI pain in an effort to improve the quality of life for patients. Although there are currently no published studies using probiotics or prebiotics to correct the GIM in patients, probiotic use in rodents has been shown to improve locomotor function and to be neuroprotective (Kigerl et al., 2016). Probiotics can also correct the urinary microbiome, offering short term improvements, but it is unclear how these changes directly affect UTI outcomes or frequency (Bossa et al., 2017). Both rodent and human SCI studies have successfully corrected the GIM using fecal transplantation (Brechmann et al., 2015). Rats receiving a fecal transplant after an SCI were found to have reduced anxiety levels, although no differences in locomotor recovery or lesion size were observed (Schmidt et al., 2020). Specialized diets, specifically low-carbohydrate/high-protein diets, have been suggested to provide benefit to patients by not only modifying the GIM composition but also improving metabolic function and pain behaviour. A study is currently underway to investigate the effect of such a diet (Yarar-Fisher et al., 2019).

## 9 Changes to the genito-urinary microbiome in spinal cord injury patients

Compared to GIM composition, which is highly dynamic and displays significant inter-individual variability, the genito-urinary microbiome is relatively stable. The male urethra is colonized with a high abundance of *Corynebacterium*, bacteria that colonize the skin near the male urethra (Lo et al., 2015). Women, on the other hand, exhibit a significant shift in the urinary microbiome composition from pre-pubescence to pubescence, and again from pubescence to menopause, where the composition returns to a similar community to pre-pubescence (Gupta et al., 2006, Zhou et al., 2007). Factors that directly alter the composition of the urinary microbiome are infections or catheterization, which are common in SCI patients both in early and later stages of injury. While the GIM is the focus of this review, a significant concern for patients with SCI is the development of neurogenic bladder (NB) after injury, a condition where nerve damage results in loss of bladder control and dysfunction (Hamid et al., 2018). It has been found that the presence of an overgrowth of bacteria in the urethra, during NB, and urinary catheterization methods can alter the healthy urinary microbiome (Fouts et al., 2012). Catheterization is also an independent risk factor for the development of UTI, with SCI patients at high risk (Esclarin De Ruz et al., 2000, Farrelly et al., 2019). SCI leads to significant changes in uroepithelial morphology as early as two hours post-injury, which results in increased urea and water permeability until 14dpi when the uroepithelia has regenerated (Apodaca et al., 2003). Although catheterization for NB after SCI is often necessary for the wellbeing of the patient, it can lead to significant changes in the urinary microbiome.

SCI patients are at high risk for infections by multi-drug resistant organisms. Therefore, it is imperative to develop strategies to control urinary tract infections. In a cohort of Canadian veterans with SCI, urinary tract infections were responsible for 51.2% of hospital visits (Guilcher et al., 2013). In healthy non-SCI patients, men and women do show the presence of different populations of bacteria: pubescent women have more *Lactobacillus* counts, and *Corynebacterium* colonizes the urethra of men. Both males and females showed a reduction in these bacteria post-injury, suggesting a departure from a normal, healthy urinary microbiome (Fouts et al., 2012, Groah et al., 2016). *Enterobacteriales* were identified as a potential major contributor to the pathogenic urine microbiome (Fouts et al., 2012). In a study including both males and females, it was found that 84.4% of patients (n = 45) were positive for *Escherichia coli* in their urine cultures. This could have been a result of reoccurring UTIs or asymptomatic bacteriuria (Dinh et al., 2019).

*Lactobacillus* species contribute to controlling the growth of more virulent bacteria (Lee et al., 2009, Fouts et al., 2012), and as such has been investigated as a potential protective treatment against UTIs. It was also found that the inflammation caused by UTI after SCI could be detected in serum with significantly elevated levels of IL-6 and IL-1RA in patients with UTIs. The elevation of these cytokines was similar to patients who had an SCI and experienced pain (Davies et al., 2007). It could be possible that the inflammation from the UTI exacerbates inflammation already present from the injury, potentially increasing the pain response. Thus, changes to the urinary microbiome can be of significant concern for SCI patients, and its effects on recovery after injury may be similar to that of the gut microbiome.

## 10 Linking the microbiome, spinal cord injury, and pain

The gut is innervated by the parasympathetic vagus nerve and sympathetic spinal nerves originating in the brainstem and spinal cord. After SCI, damaged nociceptors also innervating the gut release inflammatory mediators. This can result in neurogenic bowel, with patients experiencing constipation, fecal incontinence, and abdominal bloating and distension (Qi et al., 2018). There is mixed literature as to whether SCI affects colonic transit time. Gaudet and colleauges have demonstrated that both female and male rats receiving moderate SCI exhibit reduced whole-gut transit time 7 and 14 dpi compared to sham injured rats, with males showing reduced transit time at 43 dpi (Gaudet et al., 2019). In contrast, Jing et al. concluded that severe SCI in female mice resulted in increased whole-gut transit time 28dpi in comparison to sham injured mice (Jing et al., 2019). Some studies do suggest that increased microbial richness can help increase colonic transit time (Roager et al., 2016, Ge et al., 2017, Ding et al., 2018). Changes in gut innervation after SCI (Tottey et al., 2017, Besecker et al., 2020, Müller et al., 2020) is among the factors that may play a role in this response. Strong similarities are also observed in the frequency of abdominal pain between SCI patients and those living with chronic idiopathic constipation (Faaborg et al., 2013). However, it is difficult to determine if a neurogenic bowel and increased colonic transit time precede or is the result of GIM dysbiosis. Gut dysbiosis could exacerbate colonic transit time, further compounding abdominal pain.

A decrease in the number of bacteria producing SCFAs, namely butyrate, may lead to increased inflammation and immune cells shifting to a more proinflammatory phenotype, both of which have been associated with increased hypersensitivity after injury. Studies that have looked to correct the GIM have seen a decrease in the levels of inflammatory cytokines (Jing et al., 2019). There is a decrease in the epithelial integrity of the gut after injury, due to a decrease in tight junction proteins – as occurs in the rat bladder after SCI. Depending on the severity of injury, this could result in bacteria migrating out of the gut and interacting with sensory neurons and the immune system (Kigerl et al., 2016). This could induce a sizeable inflammatory response, nociceptor sensitization, and as a result, increased pain hypersensitivity.

GIM also plays an essential role in producing neurotransmitters. *Lactobacillus* and *Bifidobacterium*, two genera of bacteria that decrease after injury (Jing et al., 2019), play an important part in producing neurotransmitters such as serotonin, dopamine, and γ-aminobutyric acid (O'Mahony et al., 2015). Patients living with fibromyalgia, who also show gut dysbiosis, often have low levels of serotonin (Erdrich et al., 2020). Treatments that offer patients pain relief also increase serotonin levels, although it is unknown if these treatments affect the GIM (Karatay et al., 2018). Antidepressants commonly used in pain management modulate levels of neurotransmitters, including serotonin, glutamate, and γ-aminobutyric acid, which have also been observed in rodents after SCI. Thus, dysbiosis of the GIM may result in decreased neurotransmitter generation, increasing not only levels of pain but also mental health disorders seen in SCI patients.

## 11 Future directions

We are just now starting to understand the complex interactions through which the GIM can affect physiological outcomes in various models of injury and disease. Although shifts in microbial communities have been observed, a great deal more work is needed to understand how injury or disease can affect the GIM and conversely, how the GIM might affect physiological outcomes in health and disease. Many research groups are now working to study these interactions using antibiotics and/or probiotics, to determine whether and how an altered GIM may be of benefit (Ji et al., 2012, Dylag et al., 2014, Ahmad and Akbar, 2016, Kim et al., 2019). This new area of research will be key to offering potential new therapeutics for the treatment of disease. Others now focus on altering diets as an alternative to using probiotics/antibiotics to alter the GIM, a line of research that could easily be implemented in daily patient care (Singh et al., 2017, Zmora et al., 2019).

A challenge that comes with investigating the microbiome, however, is the significant variability observed between patients. The microbiome differs drastically between different races (Chen et al., 2016b) and geography (De Filippo et al., 2010), making this work even more challenging. In the search for new therapeutics, the microbiome and overall gut health should be more closely monitored, given our increased understanding of the importance of the microbiome in modulating SCI neuroimmune responses and pain. By correcting the GIM or reducing gut dysbiosis, patients could experience reduced pain, improved bowel function, and overall enriched quality of life.

## CRediT authorship contribution statement

**Courtney A. Bannerman:** Writing - original draft. **Katya Douchant:** Writing - original draft, Visualization. **Prameet M. Sheth:** Supervision, Writing - review & editing. **Nader Ghasemlou:** Supervision, Writing - review & editing.

## Declaration of Competing Interest

The authors declare that they have no known competing financial interests or personal relationships that could have appeared to influence the work reported in this paper.

## References

[b0005] Aceves M., Terminel M.N., Okoreeh A., Aceves A.R., Gong Y.M., Polanco A., Sohrabji F., Hook M.A. (2019). Morphine increases macrophages at the lesion site following spinal cord injury: Protective effects of minocycline. Brain Behav. Immun..

[b0010] Ahmad O.F., Akbar A. (2016). Microbiome, antibiotics and irritable bowel syndrome. Br. Med. Bull..

[b0015] Ahmed R.U., Alam M., Zheng Y.-P. (2019). Experimental spinal cord injury and behavioral tests in laboratory rats. Heliyon.

[b0020] Alizadeh A., Dyck S.M., Karimi-Abdolrezaee S. (2019). Traumatic Spinal Cord Injury: An Overview of Pathophysiology, Models and Acute Injury Mechanisms. Front. Neurol..

[b0025] Apodaca G., Kiss S., Ruiz W., Meyers S., Zeidel M., Birder L. (2003). Disruption of bladder epithelium barrier function after spinal cord injury. Am. J. Physiol.-Renal Physiol..

[b0030] Arora H.C., Eng C., Shoskes D.A. (2017). Gut microbiome and chronic prostatitis/chronic pelvic pain syndrome. Ann. Transl. Med..

[bib1239] Bangsgaard Bendtsen (2012). Gut microbiota composition is correlated to grid floor induced stress and behavior in the BALB/c mouse. PLoS One.

[b0035] Baral P., Umans B.D., Li L., Wallrapp A., Bist M., Kirschbaum T., Wei Y., Zhou Y., Kuchroo V.K., Burkett P.R., Yipp B.G., Liberles S.D., Chiu I.M. (2018). Nociceptor sensory neurons suppress neutrophil and gammadelta T cell responses in bacterial lung infections and lethal pneumonia. Nat. Med..

[b0040] Bastien D., Lacroix S. (2014). Cytokine pathways regulating glial and leukocyte function after spinal cord and peripheral nerve injury. Exp. Neurol..

[b0045] Bastien D., Bellver Landete V., Lessard M., Vallieres N., Champagne M., Takashima A., Tremblay M.E., Doyon Y., Lacroix S. (2015). IL-1alpha Gene Deletion Protects Oligodendrocytes after Spinal Cord Injury through Upregulation of the Survival Factor Tox3. J. Neurosci..

[b0050] Beck KD, Nguyen HX, Galvan MD, Salazar DL, Woodruff TM, Anderson AJ (2010) Quantitative analysis of cellular inflammation after traumatic spinal cord injury: evidence for a multiphasic inflammatory response in the acute to chronic environment. Brain 133, 433-447. 10.1093/brain/awp322 PMC2858013 20085927

[b0055] Behrmann DL, Bresnahan JC, Beattie MS, Shah BR (1992) Spinal cord injury produced by consistent mechanical displacement of the cord in rats: behavioral and histologic analysis. J. Neurotrauma 9, 197-217. 10.1089/neu.1992.9.197 1474608

[b0060] Belkaid Y., Harrison O.J. (2017). Homeostatic Immunity and the Microbiota. Immunity.

[b0065] Bellver-Landete V., Bretheau F., Mailhot B., Vallières N., Lessard M., Janelle M.-E., Vernoux N., Tremblay M.-È., Fuehrmann T., Shoichet M.S., Lacroix S. (2019). Microglia are an essential component of the neuroprotective scar that forms after spinal cord injury. Nat. Commun..

[b0070] Bernardi M, Fedullo AL, Bernardi E, Munzi D, Peluso I, Myers J, Lista FR, Sciarra T (2020) Diet in neurogenic bowel management: A viewpoint on spinal cord injury. World J. Gastroenterol. 26, 2479-2497. 10.3748/wjg.v26.i20.2479 PMC7265150 32523306

[b0075] Besecker E.M., Blanke E.N., Deiter G.M., Holmes G.M. (2020). Gastric vagal afferent neuropathy following experimental spinal cord injury. Exp. Neurol..

[b0080] Besecker E.M., Deiter G.M., Pironi N., Cooper T.K., Holmes G.M. (2017). Mesenteric vascular dysregulation and intestinal inflammation accompanies experimental spinal cord injury. Am. J. Physiol.-Regulat., Integrat. Comparat. Physiol..

[b0085] Boadas-Vaello P., Homs J., Portero-Tresserra M., Álvarez-Pérez B., Deulofeu M., Verdú E. (2018). Graded photochemical spinal cord injury results in chronic hyperalgesia and depression-like behaviour but no anxiety exacerbation in female BALB/c mice. Neurosci. Lett..

[b0090] Boer C.G., Radjabzadeh D., Medina-Gomez C., Garmaeva S., Schiphof D., Arp P., Koet T., Kurilshikov A., Fu J., Ikram M.A., Bierma-Zeinstra S., Uitterlinden A.G., Kraaij R., Zhernakova A., van Meurs J.B.J. (2019). Intestinal microbiome composition and its relation to joint pain and inflammation. Nat. Commun..

[b0095] Bossa L, Kline K, McDougald D, Lee BB, Rice SA (2017) Urinary catheter-associated microbiota change in accordance with treatment and infection status. PLoS One 12:e0177633. 10.1371/journal.pone.0177633 PMC5476236 28628622

[b0100] Boven LA, Van Meurs M, Van Zwam M, Wierenga-Wolf A, Hintzen RQ, Boot RG, Aerts JM, Amor S, Nieuwenhuis EE, Laman JD (2006) Myelin-laden macrophages are anti-inflammatory, consistent with foam cells in multiple sclerosis. Brain 129:517-526. 10.1093/brain/awh707 16364958

[b0105] Brechmann T., Swol J., Knop-Hammad V., Willert J., Aach M., Cruciger O., Schmiegel W., Schildhauer T.A., Hamsen U. (2015). Complicated fecal microbiota transplantation in a tetraplegic patient with severe Clostridium difficile infection. World J. Gastroenterol..

[b0110] Brenchley J.M., Price D.A., Schacker T.W., Asher T.E., Silvestri G., Rao S., Kazzaz Z., Bornstein E., Lambotte O., Altmann D., Blazar B.R., Rodriguez B., Teixeira-Johnson L., Landay A., Martin J.N., Hecht F.M., Picker L.J., Lederman M.M., Deeks S.G., Douek D.C. (2006). Microbial translocation is a cause of systemic immune activation in chronic HIV infection. Nat. Med..

[b0115] Brommer B., Engel O., Kopp M.A., Watzlawick R., Müller S., Prüss H., Chen Y., DeVivo M.J., Finkenstaedt F.W., Dirnagl U., Liebscher T., Meisel A., Schwab J.M. (2016). Spinal cord injury-induced immune deficiency syndrome enhances infection susceptibility dependent on lesion level. Brain.

[b0120] Bruce J.C., Oatway M.A., Weaver L.C. (2002). Chronic Pain after Clip-Compression Injury of the Rat Spinal Cord. Exp. Neurol..

[b0125] Bryce T.N., Biering-Sørensen F., Finnerup N.B., Cardenas D.D., Defrin R., Lundeberg T., Norrbrink C., Richards J.S., Siddall P., Stripling T., Treede R.-D., Waxman S.G., Widerström-Noga E., Yezierski R.P., Dijkers M. (2012). International Spinal Cord Injury Pain Classification: part I. Background and description. Spinal Cord.

[b0130] Burke D., Lennon O., Fullen B.M. (2018). Quality of life after spinal cord injury: The impact of pain. Eur. J. Pain.

[b0135] Burke D., Fullen B.M., Stokes D., Lennon O. (2017). Neuropathic pain prevalence following spinal cord injury: A systematic review and meta-analysis. Eur. J. Pain.

[b0140] Canani R.B., Costanzo M.D., Leone L., Pedata M., Meli R., Calignano A. (2011). Potential beneficial effects of butyrate in intestinal and extraintestinal diseases. World J. Gastroenterol..

[b0145] Carabotti M., Scirocco A., Maselli M.A., Severi C. (2015). The gut-brain axis: interactions between enteric microbiota, central and enteric nervous systems. Ann. Gastroenterol..

[b0150] Castany S., Gris G., Vela J.M., Verdú E., Boadas-Vaello P. (2018). Critical role of sigma-1 receptors in central neuropathic pain-related behaviours after mild spinal cord injury in mice. Sci. Rep..

[b0155] Chabaud M., Fossiez F., Taupin J.L., Miossec P. (1998). Enhancing effect of IL-17 on IL-1-induced IL-6 and leukemia inhibitory factor production by rheumatoid arthritis synoviocytes and its regulation by Th2 cytokines. J. Immunol..

[b0160] Chang P.V., Hao L., Offermanns S., Medzhitov R. (2014). The microbial metabolite butyrate regulates intestinal macrophage function via histone deacetylase inhibition. PNAS.

[b0165] Chen J., Chia N., Kalari K.R., Yao J.Z., Novotna M., Paz Soldan M.M., Luckey D.H., Marietta E.V., Jeraldo P.R., Chen X., Weinshenker B.G., Rodriguez M., Kantarci O.H., Nelson H., Murray J.A., Mangalam A.K. (2016). Multiple sclerosis patients have a distinct gut microbiota compared to healthy controls. Sci. Rep..

[b0170] Chen L., Zhang Y.-H., Huang T., Cai Y.-D. (2016). Gene expression profiling gut microbiota in different races of humans. Sci. Rep..

[b0175] Chen M.J., Kress B., Han X., Moll K., Peng W., Ji R.-R., Nedergaard M. (2012). Astrocytic CX43 hemichannels and gap junctions play a crucial role in development of chronic neuropathic pain following spinal cord injury. Glia.

[b0180] Chen P.S., Wang C.-C., Bortner C.D., Peng G.-S., Wu X., Pang H., Lu R.-B., Gean P.-W., Chuang D.-M., Hong J.-S. (2007). Valproic acid and other histone deacetylase inhibitors induce microglial apoptosis and attenuate lipopolysaccharide-induced dopaminergic neurotoxicity. Neuroscience.

[b0185] Cheriyan T., Ryan D.J., Weinreb J.H., Cheriyan J., Paul J.C., Lafage V., Kirsch T., Errico T.J. (2014). Spinal cord injury models: a review. Spinal Cord.

[b0190] Chhaya S.J., Quiros-Molina D., Tamashiro-Orrego A.D., Houlé J.D., Detloff M.R. (2019). Exercise-Induced Changes to the Macrophage Response in the Dorsal Root Ganglia Prevent Neuropathic Pain after Spinal Cord Injury. J. Neurotrauma.

[b0195] Chichlowski M., Rudolph C. (2015). Visceral Pain and Gastrointestinal Microbiome. J Neurogastroenterol Motil.

[b0200] Chiu I.M., Heesters B.A., Ghasemlou N., Von Hehn C.A., Zhao F., Tran J., Wainger B., Strominger A., Muralidharan S., Horswill A.R., Wardenburg J.B., Hwang S.W., Carroll M.C., Woolf C.J. (2013). Bacteria activate sensory neurons that modulate pain and inflammation. Nature.

[b0205] Cho D.C., Cheong J.H., Yang M.S., Hwang S.J., Kim J.M., Kim C.H. (2011). The Effect of Minocycline on Motor Neuron Recovery and Neuropathic Pain in a Rat Model of Spinal Cord Injury. J. Korean Neurosurg. Soc..

[b0210] Clarke D, Nguyen D, Overton K (2020) Antimicrobial stewardship in spinal cord injury: A multidisciplinary approach. J. Spinal Cord Med. :1-5. 10.1080/10790268.2020.1731225 PMC8477917 32105194

[b0215] Clarke T.B., Davis K.M., Lysenko E.S., Zhou A.Y., Yu Y., Weiser J.N. (2010). Recognition of peptidoglycan from the microbiota by Nod1 enhances systemic innate immunity. Nat. Med..

[b0220] Clos-Garcia M., Andrés-Marin N., Fernández-Eulate G., Abecia L., Lavín J.L., van Liempd S., Cabrera D., Royo F., Valero A., Errazquin N., Vega M.C.G., Govillard L., Tackett M.R., Tejada G., Gónzalez E., Anguita J., Bujanda L., Orcasitas A.M.C., Aransay A.M., Maíz O., López de Munain A., Falcón-Pérez J.M. (2019). Gut microbiome and serum metabolome analyses identify molecular biomarkers and altered glutamate metabolism in fibromyalgia. EBioMedicine.

[b0225] Craig A., Tran Y., Middleton J. (2009). Psychological morbidity and spinal cord injury: a systematic review. Spinal Cord.

[b0230] Cummings J.H. (1981). Short chain fatty acids in the human colon.. Gut.

[b0235] David S., Greenhalgh A.D., Kroner A. (2015). Macrophage and microglial plasticity in the injured spinal cord. Neuroscience.

[b0240] Davies A.L., Hayes K.C., Dekaban G.A. (2007). Clinical Correlates of Elevated Serum Concentrations of Cytokines and Autoantibodies in Patients With Spinal Cord Injury. Arch. Phys. Med. Rehabil..

[b0245] De Filippo C., Cavalieri D., Di Paola M., Ramazzotti M., Poullet J.B., Massart S., Collini S., Pieraccini G., Lionetti P. (2010). Impact of diet in shaping gut microbiota revealed by a comparative study in children from Europe and rural Africa. Proc. Natl. Acad. Sci..

[b0250] den Besten G., van Eunen K., Groen A.K., Venema K., Reijngoud D.-J., Bakker B.M. (2013). The role of short-chain fatty acids in the interplay between diet, gut microbiota, and host energy metabolism. J. Lipid Res..

[b0255] DeVivo M.J., Chen Y. (2011). Trends in New Injuries, Prevalent Cases, and Aging With Spinal Cord Injury. Arch. Phys. Med. Rehabil..

[b0260] DeVivo M.J., Kartus P.L., Stover S.L., Rutt R.D., Fine P.R. (1989). Cause of death for patients with spinal cord injuries. Arch. Intern. Med..

[b0265] Ding C, Fan W, Gu L, Tian H, Ge X, Gong J, Nie Y, Li N (2018) Outcomes and prognostic factors of fecal microbiota transplantation in patients with slow transit constipation: results from a prospective study with long-term follow-up. Gastroenterol. Rep. (Oxf) 6, pp. 101-107. 10.1093/gastro/gox036 PMC5952918 29780597

[b0270] Dinh A., Hallouin-Bernard M.C., Davido B., Lemaignen A., Bouchand F., Duran C., Even A., Denys P., Perrouin-Verbe B., Sotto A., Lavigne J.P., Bruyere F., Grall N., Tavernier E., Bernard L. (2019). Weekly sequential antibioprophylaxis for recurrent UTI among patients with neurogenic bladder: a randomized controlled trial. Clin. Infect. Dis..

[b0275] Diogenes A., Ferraz C.C.R., Akopian A.N., Henry M.A., Hargreaves K.M. (2011). LPS Sensitizes TRPV1 via Activation of TLR4 in Trigeminal Sensory Neurons. J. Dent. Res..

[b0280] Donnelly D.J., Popovich P.G. (2008). Inflammation and its role in neuroprotection, axonal regeneration and functional recovery after spinal cord injury. Exp. Neurol..

[b0285] Dumford DM, 3rd, Nerandzic M, Chang S, Richmond MA, Donskey C (2011) Epidemiology of clostridium difficile and vancomycin-resistant Enterococcus colonization in patients on a spinal cord injury unit. J. Spinal Cord Med. 34, 22-27. 10.1179/107902610x12883422813822 PMC3066494 21528623

[b0290] Duncan S.H., Louis P., Flint H.J. (2007). Cultivable bacterial diversity from the human colon. Lett. Appl. Microbiol..

[b0295] Dylag K., Hubalewska-Mazgaj M., Surmiak M., Szmyd J., Brzozowski T. (2014). Probiotics in the mechanism of protection against gut inflammation and therapy of gastrointestinal disorders. Curr. Pharm. Des..

[b0300] Erdrich S., Hawrelak J.A., Myers S.P., Harnett J.E. (2020). Determining the association between fibromyalgia, the gut microbiome and its biomarkers: A systematic review. BMC Musculoskelet. Disord..

[b0305] Esclarin De Ruz A, Garcia Leoni E, Herruzo Cabrera R (2000) Epidemiology and risk factors for urinary tract infection in patients with spinal cord injury. J. Urol. 164, 1285-1289. 10992382

[b0310] Faaborg P.M., Finnerup N.B., Christensen P., Krogh K. (2013). Abdominal Pain: A Comparison between Neurogenic Bowel Dysfunction and Chronic Idiopathic Constipation. Gastroenterol. Res. Pract..

[b0315] Failli V, Kopp MA, Gericke C, Martus P, Klingbeil S, Brommer B, Laginha I, Chen Y, DeVivo MJ, Dirnagl U, Schwab JM (2012) Functional neurological recovery after spinal cord injury is impaired in patients with infections. Brain 135:3238-3250. 10.1093/brain/aws267 23100450

[b0320] Fakhoury M. (2015). Spinal cord injury: overview of experimental approaches used to restore locomotor activity. Rev. Neurosci..

[b0325] Falnikar A., Hala T.J., Poulsen D.J., Lepore A.C. (2016). GLT1 overexpression reverses established neuropathic pain-related behavior and attenuates chronic dorsal horn neuron activation following cervical spinal cord injury: GLT1 Overexpression Reverses Neuropathic Pain-Related Behavior. Glia.

[b0330] Fandel T., Trivedi A., Nicholas C., Zhang H., Chen J., Martinez A., Noble-Haeusslein L., Kriegstein A. (2016). Transplanted Human Stem Cell-Derived Interneuron Precursors Mitigate Mouse Bladder Dysfunction and Central Neuropathic Pain after Spinal Cord Injury. Cell Stem Cell.

[b0335] Farrelly E., Lindbo L., Wijkström H., Seiger Å. (2019). The Stockholm Spinal Cord Uro Study: 1. Basic characteristics and problem inventory. Scandinav. J. Urol..

[b0340] Ferrer M., Méndez-García C., Rojo D., Barbas C., Moya A. (2017). Antibiotic use and microbiome function. Biochem. Pharmacol..

[b0345] Finnerup N.B., Norrbrink C., Trok K., Piehl F., Johannesen I.L., Sørensen J.C., Jensen T.S., Werhagen L. (2014). Phenotypes and Predictors of Pain Following Traumatic Spinal Cord Injury: A Prospective Study. J. Pain.

[b0350] Fleming JC, Norenberg MD, Ramsay DA, Dekaban GA, Marcillo AE, Saenz AD, Pasquale-Styles M, Dietrich WD, Weaver LC (2006) The cellular inflammatory response in human spinal cords after injury. Brain 129:3249-3269. 10.1093/brain/awl296 17071951

[b0355] Fouts D.E., Pieper R., Szpakowski S., Pohl H., Knoblach S., Suh M.-J., Huang S.-T., Ljungberg I., Sprague B.M., Lucas S.K., Torralba M., Nelson K.E., Groah S.L. (2012). Integrated next-generation sequencing of 16S rDNA and metaproteomics differentiate the healthy urine microbiome from asymptomatic bacteriuria in neuropathic bladder associated with spinal cord injury. J. Transl. Med..

[b0360] Fu X., Liu Z., Zhu C., Mou H., Kong Q. (2019). Nondigestible carbohydrates, butyrate, and butyrate-producing bacteria. Crit. Rev. Food Sci. Nutr..

[b0365] Fukutoku T., Kumagai G., Fujita T., Sasaki A., Wada K., Liu X., Tanaka T., Kudo H., Asari T., Nikaido Y., Ueno S., Ishibashi Y. (2020). Sex-Related Differences in Anxiety and Functional Recovery after Spinal Cord Injury in Mice. J. Neurotrauma.

[b0370] Gao J., Xu K., Liu H., Liu G., Bai M., Peng C., Li T., Yin Y. (2018). Impact of the Gut Microbiota on Intestinal Immunity Mediated by Tryptophan Metabolism. Front. Cell. Infect. Microbiol..

[b0375] Gaudet A.D., Ayala M.T., Schleicher W.E., Smith E.J., Bateman E.M., Maier S.F., Watkins L.R. (2017). Exploring acute-to-chronic neuropathic pain in rats after contusion spinal cord injury. Exp. Neurol..

[b0380] Gaudet A.D., Fonken L.K., Ayala M.T., Bateman E.M., Schleicher W.E., Smith E.J., D’Angelo H.M., Maier S.F., Watkins L.R. (2018). Spinal Cord Injury in Rats Disrupts the Circadian System. eNeuro.

[b0385] Gaudet A.D., Fonken L.K., Ayala M.T., Dangelo H.M., Smith E.J., Bateman E.M., Schleicher W.E., Maier S.F., Watkins L.R. (2019). Spinal Cord Injury in Rats Dysregulates Diurnal Rhythms of Fecal Output and Liver Metabolic Indicators. J. Neurotrauma.

[b0390] Ge X., Zhao W., Ding C., Tian H., Xu L., Wang H., Ni L., Jiang J., Gong J., Zhu W., Zhu M., Li N. (2017). Potential role of fecal microbiota from patients with slow transit constipation in the regulation of gastrointestinal motility. Sci. Rep..

[b0395] Gensel J.C., Zhang B. (2015). Macrophage activation and its role in repair and pathology after spinal cord injury. Brain Res..

[b0400] Gensel J.C., Donahue R.R., Bailey W.M., Taylor B.K. (2019). Sexual Dimorphism of Pain Control: Analgesic Effects of Pioglitazone and Azithromycin in Chronic Spinal Cord Injury. J. Neurotrauma.

[b0405] Ghasemlou N., Kerr B., David S. (2005). Tissue displacement and impact force are important contributors to outcome after spinal cord contusion injury. Exp. Neurol..

[b0410] Gill S.R., Pop M., DeBoy R.T., Eckburg P.B., Turnbaugh P.J., Samuel B.S., Gordon J.I., Relman D.A., Fraser-Liggett C.M., Nelson K.E. (2006). Metagenomic Analysis of the Human Distal Gut Microbiome. Science.

[b0415] Goodrich J., Di Rienzi S., Poole A., Koren O., Walters W., Caporaso J.G., Knight R., Ley R. (2014). Conducting a Microbiome Study. Cell.

[b0420] Goritz C., Dias D.O., Tomilin N., Barbacid M., Shupliakov O., Frisen J. (2011). A Pericyte Origin of Spinal Cord Scar Tissue. Science.

[b0425] Gorkiewicz G., Moschen A. (2018). Gut microbiome: a new player in gastrointestinal disease. Virchows Arch..

[b0430] Graham C., Mullen A., Whelan K. (2015). Obesity and the gastrointestinal microbiota: a review of associations and mechanisms. Nutr. Rev..

[b0435] Greenhalgh AD, Zarruk JG, Healy LM, Baskar Jesudasan SJ, Jhelum P, Salmon CK, Formanek A, Russo MV, Antel JP, McGavern DB, McColl BW, David S (2018) Peripherally derived macrophages modulate microglial function to reduce inflammation after CNS injury. PLoS Biol., 16:e2005264. 10.1371/journal.pbio.2005264 PMC6205650 30332405

[b0440] Groah S.L., Pérez-Losada M., Caldovic L., Ljungberg I.H., Sprague B.M., Castro-Nallar E., Chandel N.J., Hsieh M.H., Pohl H.G. (2016). Redefining Healthy Urine: A Cross-Sectional Exploratory Metagenomic Study of People With and Without Bladder Dysfunction. J. Urol..

[b0445] Gruner J.A. (1992). A Monitored Contusion Model of Spinal Cord Injury in the Rat. J. Neurotrauma.

[b0450] Guarner F., Malagelada J.-R. (2003). Gut flora in health and disease. Lancet.

[b0455] Guilcher S.J.T., Craven B.C., Calzavara A., McColl M.A., Jaglal S.B. (2013). Is the emergency department an appropriate substitute for primary care for persons with traumatic spinal cord injury?. Spinal Cord.

[b0460] Guilcher S.J.T., Hogan M.E., McCormack D., Calzavara A.J., Hitzig S.L., Patel T., Packer T., Guan Q., Lofters A.K. (2020). Prescription medications dispensed following a nontraumatic spinal cord dysfunction: a retrospective population-based study in Ontario.

[b0465] Gungor B, Adiguzel E, Gursel I, Yilmaz B, Gursel M (2016) Intestinal Microbiota in Patients with Spinal Cord Injury. PLoS One 11:e0145878. 10.1371/journal.pone.0145878 PMC4709077 26752409

[b0470] Guo R., Chen L.-H., Xing C., Liu T. (2019). Pain regulation by gut microbiota: molecular mechanisms and therapeutic potential. Br. J. Anaesth..

[b0475] Gupta S., Kumar N., Singhal N., Kaur R., Manektala U. (2006). Vaginal microflora in postmenopausal women on hormone replacement therapy. Indian J. Pathol. Microbiol..

[b0480] Guptarak J, Wanchoo S, Durham-Lee J, Wu Y, Zivadinovic D, Paulucci-Holthauzen A, Nesic O (2013) Inhibition of IL-6 signaling: A novel therapeutic approach to treating spinal cord injury pain. Pain 154:1115-1128. 10.1016/j.pain.2013.03.026 23639820

[b0485] Guzman-Rodriguez M., McDonald J.A.K., Hyde R., Allen-Vercoe E., Claud E.C., Sheth P.M., Petrof E.O. (2018). Using bioreactors to study the effects of drugs on the human microbiota. Methods.

[b0490] Hains B.C., Everhart A.W., Fullwood S.D., Hulsebosch C.E. (2002). Changes in Serotonin, Serotonin Transporter Expression and Serotonin Denervation Supersensitivity: Involvement in Chronic Central Pain after Spinal Hemisection in the Rat. Exp. Neurol..

[b0495] Hamid R., Averbeck M.A., Chiang H., Garcia A., Al Mousa R.T., Oh S.-J., Patel A., Plata M., Del Popolo G. (2018). Epidemiology and pathophysiology of neurogenic bladder after spinal cord injury. World J. Urol..

[b0500] Han T.R., Kim J.H., Kwon B.S. (1998). Chronic gastrointestinal problems and bowel dysfunction in patients with spinal cord injury. Spinal Cord.

[b0505] Herrera J., Bockhorst K., Bhattarai D., Uray K. (2020). Gastrointestinal vascular permeability changes following spinal cord injury. Neurogastroenterol. Motil..

[b0510] Holmes G.M., Blanke E.N. (2019). Gastrointestinal dysfunction after spinal cord injury. Exp. Neurol..

[b0515] Hong J, Chang A, Liu Y, Wang J, Fehlings MG (2019) Incomplete Spinal Cord Injury Reverses the Level-Dependence of Spinal Cord Injury Immune Deficiency Syndrome. Int. J. Mol. Sci. 20. 10.3390/ijms20153762 PMC6695842 31374824

[b0520] Honjoh K., Nakajima H., Hirai T., Watanabe S., Matsumine A. (2019). Relationship of Inflammatory Cytokines From M1-Type Microglia/Macrophages at the Injured Site and Lumbar Enlargement With Neuropathic Pain After Spinal Cord Injury in the CCL21 Knockout (plt) Mouse. Front. Cell. Neurosci..

[b0525] Hoschouer EL, Basso DM, Jakeman LB (2010) Aberrant sensory responses are dependent on lesion severity after spinal cord contusion injury in mice. Pain 148, 328-342. 10.1016/j.pain.2009.11.023 PMC2815266 20022699

[b0530] Hugerth L.W., Andreasson A., Talley N.J., Forsberg A.M., Kjellström L., Schmidt P.T., Agreus L., Engstrand L. (2020). No distinct microbiome signature of irritable bowel syndrome found in a Swedish random population. Gut.

[b0535] Jaglal S.B., Munce S.E.P., Guilcher S.J., Couris C.M., Fung K., Craven B.C., Verrier M. (2009). Health system factors associated with rehospitalizations after traumatic spinal cord injury: a population-based study. Spinal Cord.

[b0540] Jahromi M.S., Mure A., Gomez C.S. (2014). UTIs in Patients with Neurogenic Bladder. Curr. Urol. Rep..

[b0545] Jakeman L.B., Guan Z., WEI P., Ponnappan R., Dzwonczyk R., Popovich P.G., Stokes B.T. (2000). Traumatic Spinal Cord Injury Produced by Controlled Contusion in Mouse. J. Neurotrauma.

[b0550] Janeway C.A., Carding S., Jones B., Murray J., Portoles P., Rasmussen R., Rojo J., Saizawa K., West J., Bottomly K. (1988). CD4+ T Cells: Specificity and Function. Immunol. Rev..

[b0555] Jeffery I.B., O'Toole P.W., Öhman L., Claesson M.J., Deane J., Quigley E.M.M., Simrén M. (2012). An irritable bowel syndrome subtype defined by species-specific alterations in faecal microbiota. Gut.

[b0560] Ji J., Shu D., Zheng M., Wang J., Luo C., Wang Y., Guo F., Zou X., Lv X., Li Y., Liu T., Qu H. (2016). Microbial metabolite butyrate facilitates M2 macrophage polarization and function. Sci. Rep..

[b0565] Ji YS, Kim HN, Park HJ, Lee JE, Yeo SY, Yang JS, Park SY, Yoon HS, Cho GS, Franz CM, Bomba A, Shin HK, Holzapfel WH (2012) Modulation of the murine microbiome with a concomitant anti-obesity effect by Lactobacillus rhamnosus GG and Lactobacillus sakei NR28. Benef. Microbes 3, 13-22. 10.3920/BM2011.0046 22348905

[b0570] Jing Y., Yang D., Bai F., Zhang C., Qin C., Li D.i., Wang L., Yang M., Chen Z., Li J. (2019). Melatonin Treatment Alleviates Spinal Cord Injury-Induced Gut Dysbiosis in Mice. J. Neurotrauma.

[b0575] Kannampalli P, Shaker R, Sengupta JN (2011) Colonic butyrate- algesic or analgesic? Neurogastroenterol. Motil. 23, 975-979. 10.1111/j.1365-2982.2011.01775.x PMC3191935 21981302

[b0580] Karatay S, Okur SC, Uzkeser H, Yildirim K, Akcay F (2018) Effects of Acupuncture Treatment on Fibromyalgia Symptoms, Serotonin, and Substance P Levels: A Randomized Sham and Placebo-Controlled Clinical Trial. Pain Med. 19, 615-628. 10.1093/pm/pnx263 29220534

[b0585] Karlsson A.K. (2006). Autonomic dysfunction in spinal cord injury: clinical presentation of symptoms and signs. Prog. Brain Res..

[b0590] Kennedy P., Hasson L. (2017). The relationship between pain and mood following spinal cord injury. J. Spinal Cord Med..

[b0595] Kerr B.J., David S. (2007). Pain behaviors after spinal cord contusion injury in two commonly used mouse strains. Exp. Neurol..

[b0600] Kigerl K.A., Mostacada K., Popovich P.G. (2018). Gut Microbiota Are Disease-Modifying Factors After Traumatic Spinal Cord Injury. Neurotherapeutics.

[b0605] Kigerl K.A., Zane K., Adams K., Sullivan M.B., Popovich P.G. (2020). The spinal cord-gut-immune axis as a master regulator of health and neurological function after spinal cord injury. Exp. Neurol..

[b0610] Kigerl KA, Hall JC, Wang L, Mo X, Yu Z, Popovich PG (2016) Gut dysbiosis impairs recovery after spinal cord injury. J. Exp. Med. 213, 2603-2620. 10.1084/jem.20151345 PMC5110012 27810921

[b0615] Kim S.-K., Guevarra R.B., Kim Y.-T., Kwon J., Kim H., Cho J.H., Kim H.B., Lee J.-H. (2019). Role of Probiotics in Human Gut Microbiome-Associated Diseases. J. Microbiol. Biotechnol..

[b0620] Kjell J., Olson L. (2016). Rat models of spinal cord injury: from pathology to potential therapies. Dis. Model. Mech..

[b0625] Kleibeuker W., Gabay E., Kavelaars A., Zijlstra J., Wolf G., Ziv N., Yirmiya R., Shavit Y., Tal M., Heijnen C.J. (2008). IL-1 beta signaling is required for mechanical allodynia induced by nerve injury and for the ensuing reduction in spinal cord neuronal GRK2. Brain Behav. Immun..

[b0630] Kolstadbraaten K.M., Spreng U.J., Wisloeff‐Aase K., Gaarder C., Naess P.A., Raeder J. (2019). Incidence of chronic pain 6 y after major trauma. Acta Anaesthesiol. Scand..

[b0635] Kopp M.A., Druschel C., Meisel C., Liebscher T., Prilipp E., Watzlawick R., Cinelli P., Niedeggen A., Schaser K.-D., Wanner G.A., Curt A., Lindemann G., Nugaeva N., Fehlings M.G., Vajkoczy P., Cabraja M., Dengler J., Ertel W., Ekkernkamp A., Martus P., Volk H.-D., Unterwalder N., Kölsch U., Brommer B., Hellmann R.C., Ossami Saidy R.R., Laginha I., Prüss H., Failli V., Dirnagl U., Schwab J.M. (2013). The SCIentinel study - prospective multicenter study to define the spinal cord injury-induced immune depression syndrome (SCI-IDS) - study protocol and interim feasibility data. BMC Neurol..

[b0640] Kopper T.J., McFarlane K.E., Bailey W.M., Orr M.B., Zhang B., Gensel J.C. (2019). Delayed Azithromycin Treatment Improves Recovery After Mouse Spinal Cord Injury. Front. Cell. Neurosci..

[b0645] Kramer J.L., Minhas N.K., Jutzeler C.R., Erskine E.L., Liu L.J., Ramer M.S. (2017). Neuropathic pain following traumatic spinal cord injury: Models, measurement, and mechanisms. J. Neurosci. Res..

[b0650] Kroner A., Greenhalgh A., Zarruk J., Passos dos Santos R., Gaestel M., David S. (2014). TNF and Increased Intracellular Iron Alter Macrophage Polarization to a Detrimental M1 Phenotype in the Injured Spinal Cord. Neuron.

[b0655] Ku J.H. (2006). The management of neurogenic bladder and quality of life in spinal cord injury. BJU Int.

[b0660] Kuhn P.L., Wrathall J.R. (1998). A Mouse Model of Graded Contusive Spinal Cord Injury. J. Neurotrauma.

[b0665] Kwon B.K., Stammers A.M.T., Belanger L.M., Bernardo A., Chan D., Bishop C.M., Slobogean G.P., Zhang H., Umedaly H., Giffin M., Street J., Boyd M.C., Paquette S.J., Fisher C.G., Dvorak M.F. (2010). Cerebrospinal Fluid Inflammatory Cytokines and Biomarkers of Injury Severity in Acute Human Spinal Cord Injury. J. Neurotrauma.

[b0670] Lai N.Y., Musser M.A., Pinho-Ribeiro F.A., Baral P., Jacobson A., Ma P., Potts D.E., Chen Z., Paik D., Soualhi S., Yan Y., Misra A., Goldstein K., Lagomarsino V.N., Nordstrom A., Sivanathan K.N., Wallrapp A., Kuchroo V.K., Nowarski R., Starnbach M.N., Shi H., Surana N.K., An D., Wu C., Huh J.R., Rao M., Chiu I.M. (2020). Gut-Innervating Nociceptor Neurons Regulate Peyer’s Patch Microfold Cells and SFB Levels to Mediate Salmonella Host Defense. Cell.

[b0675] Laugerette F., Vors C., Géloën A., Chauvin M.-A., Soulage C., Lambert-Porcheron S., Peretti N., Alligier M., Burcelin R., Laville M., Vidal H., Michalski M.-C. (2011). Emulsified lipids increase endotoxemia: possible role in early postprandial low-grade inflammation. J. Nutrit. Biochem..

[b0680] Lee B.A., Leiby B.E., Marino R.J. (2016). Neurological and functional recovery after thoracic spinal cord injury. J. Spinal Cord Med..

[b0685] Lee J.W., Shim Y.H., Lee S.J. (2009). Lactobacillus colonization status in infants with urinary tract infection. Pediatr. Nephrol..

[b0690] Lerch J.K., Puga D.A., Bloom O., Popovich P.G. (2014). Glucocorticoids and macrophage migration inhibitory factor (MIF) are neuroendocrine modulators of inflammation and neuropathic pain after spinal cord injury. Semin. Immunol..

[b0695] Leung L., Cahill C.M. (2010). TNF-alpha and neuropathic pain–a review. J Neuroinflammation.

[bib1237] Li (2020). Comparison of the gut microbiome composition among individuals with acute or long-standing spinal cord injury vs. able-bodied controls. J. Spinal Cord Med..

[b0700] Li H., Kong W., Chambers C.R., Yu D., Ganea D., Tuma R.F., Ward S.J. (2018). The non-psychoactive phytocannabinoid cannabidiol (CBD) attenuates pro-inflammatory mediators, T cell infiltration, and thermal sensitivity following spinal cord injury in mice. Cell. Immunol..

[b0705] Li Y., Adamek P., Zhang H., Tatsui C.E., Rhines L.D., Mrozkova P., Li Q., Kosturakis A.K., Cassidy R.M., Harrison D.S., Cata J.P., Sapire K., Zhang H., Kennamer-Chapman R.M., Jawad A.B., Ghetti A., Yan J., Palecek J., Dougherty P.M. (2015). The Cancer Chemotherapeutic Paclitaxel Increases Human and Rodent Sensory Neuron Responses to TRPV1 by Activation of TLR4. J. Neurosci..

[b0710] Li Z., Wu F., Xu D., Zhi Z., Xu G. (2019). Inhibition of TREM1 reduces inflammation and oxidative stress after spinal cord injury (SCI) associated with HO-1 expressions. Biomed. Pharmacother..

[b0715] Lin R., Xu J., Ma Q., Chen M., Wang L., Wen S., Yang C., Ma C., Wang Y., Luo Q., Zhu N. (2020). Alterations in the fecal microbiota of patients with spinal cord injury. PLoS ONE.

[b0720] Lindsey A.E., LoVerso R.L., Tovar C.A., Hill C.E., Beattie M.S., Bresnahan J.C. (2000). An Analysis of Changes in Sensory Thresholds to Mild Tactile and Cold Stimuli after Experimental Spinal Cord Injury in the Rat. Neurorehabil. Neural. Repair.

[b0725] Liu J, An H, Jiang D, Huang W, Zou H, Meng C, Li H (2004) Study of bacterial translocation from gut after paraplegia caused by spinal cord injury in rats. Spine (Phila Pa 1976) 29:164-169. 10.1097/01.BRS.0000107234.74249.CD 14722407

[b0730] Liu S, Huang Q, He S, Chen Z, Gao X, Ma D, Duan W, Ford N, Yang F, Chen X, Raja SN, Hao D, Guan Y (2019) Dermorphin [D-Arg2, Lys4] (1-4) amide inhibits below-level heat hypersensitivity in mice after contusive thoracic spinal cord injury. Pain 160:2710-2723. 10.1097/j.pain.0000000000001671 31365470

[b0735] Liu S.Q., Ma Y.G., Peng H., Fan L. (2005). Monocyte chemoattractant protein-1 level in serum of patients with acute spinal cord injury. Chin. J. Traumatol..

[b0740] Lo S., Thiam I., Fall B., Ba-Diallo A., Diallo O.F., Diagne R., Dia M.L., Ka R., Sarr A.M., Sow A.I. (2015). Urinary tract infection with Corynebacterium aurimucosum after urethroplasty stricture of the urethra: a case report. J. Med. Case Reports.

[b0745] Ma M., Basso D.M., Walters P., Stokes B.T., Jakeman L.B. (2001). Behavioral and Histological Outcomes Following Graded Spinal Cord Contusion Injury in the C57Bl/6 Mouse. Exp. Neurol..

[b0750] Macfarlane S., Macfarlane G.T. (2003). Regulation of short-chain fatty acid production. Proc. Nutr. Soc..

[b0755] Marchand F, Tsantoulas C, Singh D, Grist J, Clark AK, Bradbury EJ, McMahon SB (2009) Effects of Etanercept and Minocycline in a rat model of spinal cord injury. Eur. J. Pain 13:673-681. 10.1016/j.ejpain.2008.08.001 18849175

[b0760] Marcondes B.F., Sreepathi S., Markowski J., Nguyen D., Stock S.R., Carvalho S., Tate D., Zafonte R., Morse L.R., Fregni F. (2016). Pain severity and mobility one year after spinal cord injury: a multicenter, cross-sectional study. Eur. J. Phys. Rehabil. Med..

[b0765] Martini A.C., Berta T., Forner S., Chen G., Bento A.F., Ji R.-R., Rae G.A. (2016). Lipoxin A4 inhibits microglial activation and reduces neuroinflammation and neuropathic pain after spinal cord hemisection. J. Neuroinflammat..

[b0770] Mattace Raso G, Simeoli R, Russo R, Iacono A, Santoro A, Paciello O, Ferrante MC, Canani RB, Calignano A, Meli R (2013) Effects of sodium butyrate and its synthetic amide derivative on liver inflammation and glucose tolerance in an animal model of steatosis induced by high fat diet. PLoS One 8:e68626. 10.1371/journal.pone.0068626 PMC3702592 23861927

[b0775] Matyas J.J., O'Driscoll C.M., Yu L., Coll-Miro M., Daugherty S., Renn C.L., Faden A.I., Dorsey S.G., Wu J. (2017). Truncated TrkB.T1-Mediated Astrocyte Dysfunction Contributes to Impaired Motor Function and Neuropathic Pain after Spinal Cord Injury. J. Neurosci..

[b0780] McFarlane K., Otto T.E., Bailey W.M., Veldhorst A.K., Donahue R.R., Taylor B.K., Gensel J.C. (2020). Effect of Sex on Motor Function, Lesion Size, and Neuropathic Pain after Contusion Spinal Cord Injury in Mice. J. Neurotrauma.

[b0785] Meehan CJ, Beiko RG (2014) A phylogenomic view of ecological specialization in the Lachnospiraceae, a family of digestive tract-associated bacteria. Genome Biol. Evol. 6:703-713. 10.1093/gbe/evu050 PMC3971600 24625961

[b0790] Mehta S, McIntyre A, Janzen S, Loh E, Teasell R, Spinal Cord Injury Rehabilitation Evidence T (2016) Systematic Review of Pharmacologic Treatments of Pain After Spinal Cord Injury: An Update. Arch. Phys. Med. Rehabil. 97:1381-1391 e1381. 10.1016/j.apmr.2015.12.023 26797114

[b0795] Meisner J.G., Marsh A.D., Marsh D.R. (2010). Loss of GABAergic Interneurons in Laminae I–III of the Spinal Cord Dorsal Horn Contributes to Reduced GABAergic Tone and Neuropathic Pain after Spinal Cord Injury. J. Neurotrauma.

[b0800] Meseguer V., Alpizar Y.A., Luis E., Tajada S., Denlinger B., Fajardo O., Manenschijn J.-A., Fernández-Peña C., Talavera A., Kichko T., Navia B., Sánchez A., Señarís R., Reeh P., Pérez-García María.T., López-López José.Ramón., Voets T., Belmonte C., Talavera K., Viana Félix (2014). TRPA1 channels mediate acute neurogenic inflammation and pain produced by bacterial endotoxins. Nat. Commun..

[b0805] Middleton J.W., Lim K., Taylor L., Soden R., Rutkowski S. (2004). Patterns of morbidity and rehospitalisation following spinal cord injury. Spinal Cord.

[b0810] Mirza A., Mao-Draayer Y. (2017). The gut microbiome and microbial translocation in multiple sclerosis. Clin. Immunol..

[b0815] Miyake S, Kim S, Suda W, Oshima K, Nakamura M, Matsuoka T, Chihara N, Tomita A, Sato W, Kim SW, Morita H, Hattori M, Yamamura T (2015) Dysbiosis in the Gut Microbiota of Patients with Multiple Sclerosis, with a Striking Depletion of Species Belonging to Clostridia XIVa and IV Clusters. PLoS One 10:e0137429. 10.1371/journal.pone.0137429 PMC4569432 26367776

[b0820] Moloney R.D., Johnson A.C., O'Mahony S.M., Dinan T.G., Greenwood-Van Meerveld B., Cryan J.F. (2016). Stress and the Microbiota-Gut-Brain Axis in Visceral Pain: Relevance to Irritable Bowel Syndrome. CNS Neurosci. Ther..

[b0825] Morrison D.J., Preston T. (2016). Formation of short chain fatty acids by the gut microbiota and their impact on human metabolism. Gut Microbes.

[b0830] Muller M, Hermes GDA, Canfora EE, Smidt H, Masclee AAM, Zoetendal EG, Blaak EE (2020) Distal colonic transit is linked to gut microbiota diversity and microbial fermentation in humans with slow colonic transit. Am. J. Physiol. Gastrointest. Liver Physiol. 318, G361-G369. 10.1152/ajpgi.00283.2019 31869241

[b0835] Muller R, Landmann G, Bechir M, Hinrichs T, Arnet U, Jordan X, Brinkhof MWG (2017) Chronic pain, depression and quality of life in individuals with spinal cord injury: Mediating role of participation. J. Rehabil. Med. 49, 489-496. 10.2340/16501977-2241 28597908

[b0840] Myers S.A., Gobejishvili L., Saraswat Ohri S., Garrett Wilson C., Andres K.R., Riegler A.S., Donde H., Joshi-Barve S., Barve S., Whittemore S.R. (2019). Following spinal cord injury, PDE4B drives an acute, local inflammatory response and a chronic, systemic response exacerbated by gut dysbiosis and endotoxemia. Neurobiol. Dis..

[b0845] Mylotte JM, Graham R, Kahler L, Young L, Goodnough S (2000) Epidemiology of nosocomial infection and resistant organisms in patients admitted for the first time to an acute rehabilitation unit. Clin. Infect. Dis. 30, 425-432. 10.1086/313708 10722423

[b0850] Nagy-Szakal D., Williams B.L., Mishra N., Che X., Lee B., Bateman L., Klimas N.G., Komaroff A.L., Levine S., Montoya J.G., Peterson D.L., Ramanan D., Jain K., Eddy M.L., Hornig M., Lipkin W.I. (2017). Fecal metagenomic profiles in subgroups of patients with myalgic encephalomyelitis/chronic fatigue syndrome. Microbiome.

[bib1241] Nally (2018). Identification of *Burkholderia fungorum* in the urine of an individual with spinal cord injury and augmentation cystoplasty using 16S sequencing: copathogen or innocent bystander?. Spinal Cord Ser. Cases.

[b0855] Naparstek Y., Cohen I.R., Fuks Z., Vlodavsky I. (1984). Activated T lymphocytes produce a matrix-degrading heparan sulphate endoglycosidase. Nature.

[b0860] Nash M.S., Gater D.R. (2020). Cardiometabolic Disease and Dysfunction Following Spinal Cord Injury. Phys. Med. Rehabilit. Clin. North Am..

[b0865] Nees TA, Tappe-Theodor A, Sliwinski C, Motsch M, Rupp R, Kuner R, Weidner N, Blesch A (2016) Early-onset treadmill training reduces mechanical allodynia and modulates calcitonin gene-related peptide fiber density in lamina III/IV in a mouse model of spinal cord contusion injury. Pain 157:687-697. 10.1097/j.pain.0000000000000422 26588690

[b0870] Nielsen S.D., Faaborg P.M., Christensen P., Krogh K., Finnerup N.B. (2017). Chronic abdominal pain in long-term spinal cord injury: a follow-up study. Spinal Cord.

[b0875] Nseyo U., Santiago-Lastra Y. (2017). Long-Term Complications of the Neurogenic Bladder. Urol. Clin. North Am..

[b0880] O'Connor G., Jeffrey E., Madorma D., Marcillo A., Abreu M.T., Deo S.K., Dietrich W.D., Daunert S. (2018). Investigation of Microbiota Alterations and Intestinal Inflammation Post-Spinal Cord Injury in Rat Model. J. Neurotrauma.

[b0885] O’Mahony S.M., Clarke G., Borre Y.E., Dinan T.G., Cryan J.F. (2015). Serotonin, tryptophan metabolism and the brain-gut-microbiome axis. Behav. Brain Res..

[b0890] Oliphant K., Allen-Vercoe E. (2019). Macronutrient metabolism by the human gut microbiome: major fermentation by-products and their impact on host health. Microbiome.

[b0895] Orr M.B., Gensel J.C. (2018). Spinal Cord Injury Scarring and Inflammation: Therapies Targeting Glial and Inflammatory Responses. Neurotherapeutics.

[b0900] Pan Y., Liu B., Li R., Zhang Z., Lu L. (2014). Bowel Dysfunction in Spinal Cord Injury: Current Perspectives. Cell Biochem. Biophys..

[b0905] Park J., Decker J.T., Smith D.R., Cummings B.J., Anderson A.J., Shea L.D. (2018). Reducing inflammation through delivery of lentivirus encoding for anti-inflammatory cytokines attenuates neuropathic pain after spinal cord injury. J. Control. Release.

[b0910] Pineau I., Lacroix S. (2007). Proinflammatory cytokine synthesis in the injured mouse spinal cord: Multiphasic expression pattern and identification of the cell types involved. J. Comp. Neurol..

[b0915] Pinho-Ribeiro F.A., Verri W.A., Chiu I.M. (2017). Nociceptor Sensory Neuron–Immune Interactions in Pain and Inflammation. Trends Immunol..

[bib1236] Pires (2016). Alterations in the genital microbiota in women with spinal cord injury. Obstet Gynecol..

[b0920] Qi Z., Middleton J.W., Malcolm A. (2018). Bowel Dysfunction in Spinal Cord Injury. Curr. Gastroenterol. Rep..

[b0925] Qian J., Zhu W., Lu M., Ni B., Yang J. (2017). D-beta-hydroxybutyrate promotes functional recovery and relieves pain hypersensitivity in mice with spinal cord injury. Br. J. Pharmacol..

[b0930] Qualls-Creekmore E., Tong M., Holmes G.M. (2010). Time-course of recovery of gastric emptying and motility in rats with experimental spinal cord injury. Neurogastroenterol. Motil..

[b0935] Raja S.N., Carr D.B., Cohen M., Finnerup N.B., Flor H., Gibson S., Keefe F.J., Mogil J.S., Ringkamp M., Sluka K.A., Song X.-J., Stevens B., Sullivan M.D., Tutelman P.R., Ushida T., Vader K. (2020). The revised International Association for the Study of Pain definition of pain: concepts, challenges, and compromises. Pain.

[b0940] Raoof R, Willemen H, Eijkelkamp N (2018) Divergent roles of immune cells and their mediators in pain. Rheumatology (Oxford) 57:429-440. 10.1093/rheumatology/kex308 PMC5850827 28968842

[b0945] Reichenberger E.R., Alexander G.M., Perreault M.J., Russell J.A., Schwartzman R.J., Hershberg U., Rosen G. (2013). Establishing a relationship between bacteria in the human gut and Complex Regional Pain Syndrome. Brain Behav. Immun..

[b0950] Ren K., Dubner R. (2010). Interactions between the immune and nervous systems in pain. Nat. Med..

[b0955] Riegger T., Conrad S., Schluesener H.J., Kaps H.-P., Badke A., Baron C., Gerstein J., Dietz K., Abdizahdeh M., Schwab J.M. (2009). Immune depression syndrome following human spinal cord injury (SCI): A pilot study. Neuroscience.

[b0960] Rios-Covian D., Ruas-Madiedo P., Margolles A., Gueimonde M., de Los Reyes-Gavilan C.G., Salazar N. (2016). Intestinal Short Chain Fatty Acids and their Link with Diet and Human Health. Front. Microbiol..

[b0965] Rizzetto L., Fava F., Tuohy K.M., Selmi C. (2018). Connecting the immune system, systemic chronic inflammation and the gut microbiome: The role of sex. J. Autoimmun..

[b0970] Roager HM, Hansen LB, Bahl MI, Frandsen HL, Carvalho V, Gobel RJ, Dalgaard MD, Plichta DR, Sparholt MH, Vestergaard H, Hansen T, Sicheritz-Ponten T, Nielsen HB, Pedersen O, Lauritzen L, Kristensen M, Gupta R, Licht TR (2016) Colonic transit time is related to bacterial metabolism and mucosal turnover in the gut. Nat. Microbiol. 1, 16093. 10.1038/nmicrobiol.2016.93 27562254

[b0975] Romo P.G.B., Smith C.P., Cox A., Averbeck Márcio.A., Dowling C., Beckford C., Manohar P., Duran S., Cameron A.P. (2018). Non-surgical urologic management of neurogenic bladder after spinal cord injury. World J. Urol..

[b0980] Rosser E.C., Mauri C. (2016). A clinical update on the significance of the gut microbiota in systemic autoimmunity. J. Autoimmun..

[b0985] Russo R., Cristiano C., Avagliano C., De Caro C., La Rana G., Raso G.M., Canani R.B., Meli R., Calignano A. (2018). Gut-brain Axis: Role of Lipids in the Regulation of Inflammation, Pain and CNS Diseases. CMC.

[b0990] Saiwai H., Ohkawa Y., Yamada H., Kumamaru H., Harada A., Okano H., Yokomizo T., Iwamoto Y., Okada S. (2010). The LTB4-BLT1 Axis Mediates Neutrophil Infiltration and Secondary Injury in Experimental Spinal Cord Injury. Am. J. Pathol..

[b0995] Saria A (1984) Substance P in sensory nerve fibres contributes to the development of oedema in the rat hind paw after thermal injury. Br. J. Pharmacol. 82:217-222. 10.1111/j.1476-5381.1984.tb16461.x PMC1987261 6203590

[b1000] Sauer SK, Reeh PW, Bove GM (2001) Noxious heat-induced CGRP release from rat sciatic nerve axons in vitro. Eur. J. Neurosci. 14, 1203-1208. 10.1046/j.0953-816x.2001.01741.x 11703449

[b1005] Scheff S.W., Rabchevsky A.G., Fugaccia I., Main J.A., Lumpp J.E. (2003). Experimental Modeling of Spinal Cord Injury: Characterization of a Force-Defined Injury Device. J. Neurotrauma.

[b1010] Schmidt EKA, Torres-Espin A, Raposo PJF, Madsen KL, Kigerl KA, Popovich PG, Fenrich KK, Fouad K (2020) Fecal transplant prevents gut dysbiosis and anxiety-like behaviour after spinal cord injury in rats. PLoS One 15:e0226128. 10.1371/journal.pone.0226128 PMC6961833 31940312

[b1015] Scholz J., Woolf C.J. (2007). The neuropathic pain triad: neurons, immune cells and glia. Nat. Neurosci..

[b1020] Sekhon LH, Fehlings MG (2001) Epidemiology, demographics, and pathophysiology of acute spinal cord injury. Spine (Phila Pa 1976) 26:S2-12. 10.1097/00007632-200112151-00002 11805601

[b1025] Sencar L., Yilmaz Derviş.M., Tuli A., Polat S. (2020). Effects of combined treatment of minocycline and methylprednisolone on the expression of tumor necrosis factor alpha and interleukine-6 in experimental spinal cord injury: a light and electron microscopic study. Ultrastruct. Pathol..

[b1030] Sezer N., Akkus S., Ugurlu F.G. (2015). Chronic complications of spinal cord injury. World J Orthop.

[b1035] Shapouri-Moghaddam A., Mohammadian S., Vazini H., Taghadosi M., Esmaeili S.-A., Mardani F., Seifi B., Mohammadi A., Afshari J.T., Sahebkar A. (2018). Macrophage plasticity, polarization, and function in health and disease. J. Cell. Physiol..

[bib1240] Sharon (2014). Specialized metabolites from the microbiome in health and disease. Cell Metab..

[b1040] Shen S., Lim G., You Z., Ding W., Huang P., Ran C., Doheny J., Caravan P., Tate S., Hu K., Kim H., McCabe M., Huang B., Xie Z., Kwon D., Chen L., Mao J. (2017). Gut microbiota is critical for the induction of chemotherapy-induced pain. Nat. Neurosci..

[b1045] Shi N., Li N., Duan X., Niu H. (2017). Interaction between the gut microbiome and mucosal immune system. Military Med. Res..

[b1050] Shin J.E., Jung K., Kim M., Hwang K., Lee H., Kim I.-S., Lee B.H., Lee I.-S., Park K.I. (2018). Brain and spinal cord injury repair by implantation of human neural progenitor cells seeded onto polymer scaffolds. Exp. Mol. Med..

[b1055] Siddall P.J., Taylor D.A., Cousins M.J. (1997). Classification of pain following spinal cord injury. Spinal Cord.

[b1060] Siddall PJ, McClelland JM, Rutkowski SB, Cousins MJ (2003) A longitudinal study of the prevalence and characteristics of pain in the first 5 years following spinal cord injury. Pain 103:249-257. 10.1016/S0304-3959(02)00452-9 12791431

[b1065] Singh R.K., Chang H.-W., Yan D., Lee K.M., Ucmak D., Wong K., Abrouk M., Farahnik B., Nakamura M., Zhu T.H., Bhutani T., Liao W. (2017). Influence of diet on the gut microbiome and implications for human health. J Transl Med.

[b1070] Soden R.J., Walsh J., Middleton J.W., Craven M.L., Rutkowski S.B., Yeo J.D. (2000). Causes of death after spinal cord injury. Spinal Cord.

[b1075] Sokol H, Seksik P, Furet JP, Firmesse O, Nion-Larmurier I, Beaugerie L, Cosnes J, Corthier G, Marteau P, Dore J (2009) Low counts of Faecalibacterium prausnitzii in colitis microbiota. Inflamm. Bowel Dis. 15, 1183-1189. 10.1002/ibd.20903 19235886

[b1080] Sroga J.M., Jones T.B., Kigerl K.A., McGaughy V.M., Popovich P.G. (2003). Rats and mice exhibit distinct inflammatory reactions after spinal cord injury. J. Comp. Neurol..

[b1085] Steward O., Willenberg R. (2017). Rodent spinal cord injury models for studies of axon regeneration. Exp. Neurol..

[b1090] Stone J.M., Nino-Murcia M., Wolfe V.A., Perkash I. (1990). Chronic gastrointestinal problems in spinal cord injury patients: a prospective analysis. Am. J. Gastroenterol..

[b1095] Sun G., Yang S., Cao G., Wang Q., Hao J., Wen Q., Li Z., So K.F., Liu Z., Zhou S., Zhao Y., Yang H., Zhou L., Yin Z. (2018). gammadelta T cells provide the early source of IFN-gamma to aggravate lesions in spinal cord injury. J. Exp. Med..

[b1100] Takaki M., Mawe G.M., Barasch J.M., Gershon M.D., Gershon M.D. (1985). Physiological responses of guinea-pig myenteric neurons secondary to the release of endogenous serotonin by tryptamine. Neuroscience.

[b1105] Tateda S., Kanno H., Ozawa H., Sekiguchi A., Yahata K., Yamaya S., Itoi E. (2017). Rapamycin suppresses microglial activation and reduces the development of neuropathic pain after spinal cord injury. J. Orthop. Res..

[b1110] Toh S.-L., Lee B.B., Simpson J.M., Rice S.A., Kotsiou G., Marial O., Ryan S. (2020). Effect of probiotics on multi-resistant organism colonisation in persons with spinal cord injury: secondary outcome of ProSCIUTTU, a randomised placebo-controlled trial. Spinal Cord.

[b1115] Tottey W., Feria-Gervasio D., Gaci N., Laillet B., Pujos E., Martin J.-F., Sebedio J.-L., Sion B., Jarrige J.-F., Alric M., Brugère J.-F. (2017). Colonic Transit Time Is a Driven Force of the Gut Microbiota Composition and Metabolism: In Vitro Evidence. J. Neurogastroenterol. Motil..

[b1120] van Gorp S., Kessels A.G., Joosten E.A., van Kleef M., Patijn J. (2015). Pain prevalence and its determinants after spinal cord injury: A systematic review: Spinal cord injury pain determinants. EJP.

[b1125] Verma V., Sheikh Z., Ahmed A.S. (2015). Nociception and role of immune system in pain. Acta Neurol. Belg..

[b1130] Vierck C. (2019). Mechanisms of Below-Level Pain Following Spinal Cord Injury (SCI). J. Pain.

[b1135] Wadachi R., Hargreaves K.M. (2006). Trigeminal Nociceptors Express TLR-4 and CD14: a Mechanism for Pain due to Infection. J. Dent. Res..

[b1140] Wagner BD, Grunwald GK, Zerbe GO, Mikulich-Gilbertson SK, Robertson CE, Zemanick ET, Harris JK (2018) On the Use of Diversity Measures in Longitudinal Sequencing Studies of Microbial Communities. Front. Microbiol. 9, 1037. 10.3389/fmicb.2018.01037 PMC5972327 29872428

[b1145] Walker C.L., Fry C.M.E., Wang J., Du X., Zuzzio K., Liu N.-K., Walker M.J., Xu X.-M. (2019). Functional and Histological Gender Comparison of Age-Matched Rats after Moderate Thoracic Contusive Spinal Cord Injury. J. Neurotrauma.

[b1150] Wallace DJ, Sayre NL, Patterson TT, Nicholson SE, Hilton D, Grandhi R (2019) Spinal cord injury and the human microbiome: beyond the brain-gut axis. Neurosurg Focus 46:E11. 10.3171/2018.12.FOCUS18206 30835680

[b1155] Wang Y., Xu X., Hu P., Jia N., Ji S., Yuan H. (2019). Effect of Toll-Like Receptor 4/Myeloid Differentiation Factor 88 Inhibition by Salvianolic Acid B on Neuropathic Pain After Spinal Cord Injury in Mice. World Neurosurgery.

[b1160] Watanabe S., Uchida K., Nakajima H., Matsuo H., Sugita D., Yoshida A., Honjoh K., Johnson W.E.B., Baba H. (2015). Early Transplantation of Mesenchymal Stem Cells After Spinal Cord Injury Relieves Pain Hypersensitivity Through Suppression of Pain-Related Signaling Cascades and Reduced Inflammatory Cell Recruitment: Transplanted MSC Reduce Hypersensitivity After SCI. Stem Cells.

[b1165] Woolf C.J. (2010). What is this thing called pain?. J. Clin. Invest..

[b1170] Wu X., Liu J., Tanadini L.G., Lammertse D.P., Blight A.R., Kramer J.L.K., Scivoletto G., Jones L., Kirshblum S., Abel R., Fawcett J., Field-Fote E., Guest J., Levinson B., Maier D., Tansey K., Weidner N., Tetzlaff W.G., Hothorn T., Curt A., Steeves J.D. (2015). Challenges for defining minimal clinically important difference (MCID) after spinal cord injury. Spinal Cord.

[b1175] Wynn T.A., Vannella K.M. (2016). Macrophages in Tissue Repair, Regeneration, and Fibrosis. Immunity.

[b1180] Xiang H.C., Lin L.X., Hu X.F., Zhu H., Li H.P., Zhang R.Y., Hu L., Liu W.T., Zhao Y.L., Shu Y., Pan H.L., Li M. (2019). AMPK activation attenuates inflammatory pain through inhibiting NF-kappaB activation and IL-1beta expression. J. Neuroinflammat..

[b1185] Yang L, Blumbergs PC, Jones NR, Manavis J, Sarvestani GT, Ghabriel MN (2004) Early expression and cellular localization of proinflammatory cytokines interleukin-1beta, interleukin-6, and tumor necrosis factor-alpha in human traumatic spinal cord injury. Spine (Phila Pa 1976) 29:966-971. 10.1097/00007632-200405010-00004 15105666

[b1190] Yarar-Fisher C., Li J., McLain A., Gower B., Oster R., Morrow C. (2019). Utilizing a low-carbohydrate/high-protein diet to improve metabolic health in individuals with spinal cord injury (DISH): study protocol for a randomized controlled trial. Trials.

[b1195] Yarom Y., Naparstek Y., Lev-Ram V., Holoshitz J., Ben-Nun A., Cohen I.R. (1983). Immunospecific inhibition of nerve conduction by T lymphocytes reactive to basic protein of myelin. Nature.

[b1200] You H.-J., Colpaert F.C., Arendt-Nielsen L. (2008). Long-lasting descending and transitory short-term spinal controls on deep spinal dorsal horn nociceptive-specific neurons in response to persistent nociception. Brain Res. Bull..

[b1205] You T., Bi Y., li J., Zhang M., Chen X., Zhang K., Li J. (2017). IL-17 induces reactive astrocytes and up-regulation of vascular endothelial growth factor (VEGF) through JAK/STAT signaling. Sci. Rep..

[bib1238] Yu (2017). Variations in gut microbiota and fecal metabolic phenotype associated with depression by 16S rRNA gene sequencing and LC/MS-based metabolomics. J. Pharm. Biomed. Anal..

[b1210] Zeng Y., Han H., Tang B., Chen J., Mao D., Xiong M. (2018). Transplantation of Recombinant Vascular Endothelial Growth Factor (VEGF)189-Neural Stem Cells Downregulates Transient Receptor Potential Vanilloid 1 (TRPV1) and Improves Motor Outcome in Spinal Cord Injury. Med. Sci. Monit..

[b1215] Zhang C., Zhang W., Zhang J., Jing Y., Yang M., Du L., Gao F., Gong H., Chen L., Li J., Liu H., Qin C., Jia Y., Qiao J., Wei B., Yu Y., Zhou H., Liu Z., Yang D., Li J. (2018). Gut microbiota dysbiosis in male patients with chronic traumatic complete spinal cord injury. J. Transl. Med..

[b1220] Zhang C., Jing Y., Zhang W., Zhang J., Yang M., Du L., Jia Y., Chen L., Gong H., Li J., Gao F., Liu H., Qin C., Liu C., Wang Y., Shi W., Zhou H., Liu Z., Yang D., Li J. (2019). Dysbiosis of gut microbiota is associated with serum lipid profiles in male patients with chronic traumatic cervical spinal cord injury. Am. J. Transl. Res..

[b1225] Zhou X., Brown C.J., Abdo Z., Davis C.C., Hansmann M.A., Joyce P., Foster J.A., Forney L.J. (2007). Differences in the composition of vaginal microbial communities found in healthy Caucasian and black women. ISME J..

[b1230] Zmora N., Suez J., Elinav E. (2019). You are what you eat: diet, health and the gut microbiota. Nat. Rev. Gastroenterol. Hepatol..

[b1235] Zong S., Zeng G., Fang Y., Peng J., Tao Y., Li K., Zhao J. (2014). The Role of IL-17 Promotes Spinal Cord Neuroinflammation via Activation of the Transcription Factor STAT3 after Spinal Cord Injury in the Rat. Mediators Inflamm..

